# Supplementary material for: Combining machine learning and conventional statistical approaches for risk factor discovery in a large cohort study
Source: Sci Rep. 2021 Nov 26;11:22997. doi: 10.1038/s41598-021-02476-9 (PMC8626442; doi:10.1038/s41598-021-02476-9)
Supplement: Supplementary file 1 — Supplementary Information. [file 41598_2021_2476_MOESM1_ESM.docx]

**Combining machine learning and conventional statistical approaches for risk factor discovery in a large cohort study**

Iqbal Madakkatel*, PhD^1,2^, Ang Zhou, PhD^1,3^, Mark D. McDonnell^†^, associate professor, PhD^2^, Elina Hyppönen*^†^, professor, PhD^1,3^

^1^ Australian Centre for Precision Health, UniSA Clinical & Health Sciences, University of South Australia, Adelaide, Australia

^2^ Computational Learning Systems Laboratory, UniSA STEM, University of South Australia, Mawson Lakes, Australia

^3^ South Australian Health and Medical Research Institute, Adelaide, Australia

^†^Joint senior authors

**Supplementary Material**

**Contents**

| **Supplementary Note** | 3 |
| --- | --- |
| **Supplementary Methods** | 5 |
| **Supplementary Table S1** | 13 |
| **Supplementary Table S2** | 18 |
| **Supplementary Table S3** | 19 |
| **Supplementary Table S4** | 21 |
| **Supplementary Table S5** | 26 |
| **Supplementary Figure S1** | 35 |
| **Supplementary Figure S2** | 36 |
| **Supplementary Figure S3** | 37 |
| **Supplementary Figure S4** | 41 |
| **Supplementary Figure S5** | 46 |
| **Supplementary Figure S6** | 47 |
| **Supplementary Figure S7** | 48 |

**SUPPLEMENTARY NOTE**

Here we provide notes on the machine learning algorithms which have been found outperforming traditional risk scoring systems for mortality prediction. We exclude gradient boosting decision trees (GBDT) from these notes as they are discussed in detail in Supplementary Methods.

**Support Vector Machine (SVM)**

SVM^1^ is one of the popular supervised machine learning algorithms. When they were introduced in 1990s, they were very popular and were considered the go-to method for classification and regression. The idea behind SVM is to create a classifier that maximizes the distance (also known as margin) from closest samples to the hyperplane that separates samples of different classes. When an SVM classifier is trained, it creates a hyperplane or set of hyperplanes in high- or infinite-dimensional space. Often, the training data is noisy with outliers and mis-labeled data and one may have to resort to soft margin, which allows misclassification of some samples (within the margin) in order to reduce variance of models developed. Cross-validation can be used to identify appropriate soft margin. When data cannot be linearly separated, one can try SVM with non-linear kernels such as polynomial or radial basis function kernels.

**K-nearest neighbor (KNN)**

KNN is a non-parametric, instance-based learning (also called memory-based learning) method for classification or regression, developed in 1950s^2^. There are no models *per se* in KNN, but to store the entire training set and postpone the computation until a test instance becomes available. For classification, the class of a test instance is determined by plurality of vote of the K-closest training samples (they could be identified using distance measures such as Euclidean distance) of the test instance. Other popular distance metrics that could be used are hamming distance, Manhattan distance, and Makowski distance. K is usually an integer and a small number, a hyper-parameter of the KNN algorithm. If KNN is used for regression, then the average value of the output of the K-nearest neighbors is reported as the predicted value for the test instance.

**Artificial neural network (ANN)**

ANNs are powerful machine learning algorithms inspired by biological neural networks that can be used for classification and regression. ANN has its root in the preliminary works by McCulloch and Pitts in 1940s ^3^. The oldest neural network, the perceptron was created by Rosenblatt in 1958 ^4^. An ANN model contains connected layers of nodes (aka neurons). A neuron receives inputs from other neurons or from outside, processes them and signals other neurons or produces some output. The processing is usually non-linear in nature and a neuron signals other neurons when the value from processing exceeds some threshold. The connections between the nodes are called edges. Each edge typically will have a weight (a real number) and is adjusted during the training, typically using the backpropagation algorithm. The layers of nodes that can be found in a typical ANN are an input layer, one or more hidden layers and one output layer. Usually, neural networks are classified into three subtypes, namely, feedforward neural networks or multi-layer perceptrons (MLPs), convolutional neural networks (CNNs) and recurrent neural networks (RNNs). A neural network that consists of more than three layers (including input, hidden and output layers) is also known as a deep neural network.

**SUPPLEMENTARY METHODS**

**Background of GBDT**

Gradient boosting decision trees (GBDT) are ensembles of decision trees that are constructed in a sequence such that each subsequent tree after the first tree is trained to predict the error (also called pseudo-residuals) between the observed and predicted value obtained to that point. They are trained using supervised learning. Each decision tree is built by splitting the entire training samples into smaller and smaller groups successively until a predefined condition such as depth of the tree (as determined using a hyper-parameter) or other termination conditions are met. Each training sample will belong to only one leaf of a tree and, in our application, each leaf predicts some level of mortality. Predictors and their values at which the split is to be made to create two new branches to successively build a tree, are chosen by the algorithm in order to optimize an objective function. A development set can be used to avoid overfitting by limiting the number of such decision trees created. GBDTs are currently considered to be the state-of-the-art supervised learning algorithm for building predictive models using tabular data. They have been shown to outperform other machine learning (ML) and traditional statistical methods for mortality predictions in various settings ^5-8^. In a more general classification setting, a study conducted to assess the performance of 13 state-of-the-art ML algorithms on a set of 165 publicly available classification problems (mostly bioinformatics problems) found GBDT to be the top ranked in mean ranking across the problems ^9^. There are end-to-end implementations of GBDTs, capable of handling billions of samples and millions of variables such as XGBoost, LightGBM, and CatBoost. They utilize graphical processing units (GPUs) in addition to central processing units to improve training and inference speed.

Each ML algorithm has its own hyperparameters, also known as tuning parameters and that can control the behavior of an algorithm. Examples of such hyperparameters include learning rate, depth of decision trees, maximum number of decision trees to be created and so on. Generally, CatBo[ost](#_bookmark6) ^10^ outperforms XGBoost and LightGBM in performance using default hyperparameters ^11,12^. Also, improvements in performance using tuned hyperparameters may be very small as compared to performance with default hyperparameters as seen in some studies using CatBoost ^12^. CatBoost provides native support to handle categorical predictors (with or without numeric values). It can be instructed to consider missing value as an instance of value, guaranteeing a split between missing value and other non-missing values while trees are built.

**Specific GBDT methods**

For CatBoost, the most important hyperparameters are learning rate and number of trees to be built (also known as number of iterations or number of estimators). If used with default values, CatBoost dynamically selects learning rate based on number of iterations (default value of iterations is 1,000). For our experiments, we set the number of iterations to 10,000 and used a development set to avoid overfitting by stopping growing new trees when there is no improvement in area under the receiver operating characteristics curve (AUROC) performance on the development set in 50 consecutive iterations. Models were set to utilize GPUs and boosting type was set to plain boosting. All other hyper-parameters were left at their default values. We used predictors without imputing missing values as we used only baseline predictors (which were available for at least 95% of the participants) and hospital diagnoses.

**Pre-processing using PHESANT**

PHESANT (PHEnome Scan Analysis) ^13^ classifies variables as continuous, ordinal, and categorical using a rule-based system to determine the appropriate coding of each variable. It also deals with various scenarios such as handling of multiple initial measurement of variables (e.g. spirometry), coding unusual values (e.g. negative values are used to code answers such as ‘Preferred not to answer’ and ‘Don’t know’) as missing, changing the order of variable values to make them logical (e.g., field 1239, current smoking status) and creating proper dummy variables (e.g., secondary diagnoses field 41204 had 184 array elements and any ICD10 code could be stored in any array elements).

**SHAP (SHapley Additive exPlanation) Values**

SHAP values are based on Shapley values (derived by Lloyd Shapley in 1953 ^14^), a solution concept in game theory. Shapley values deal with how fairly (by satisfying certain conditions) the payoffs from a cooperative game can be distributed to game players. Shapley value for a player is defined as the average marginal contribution of that player, considering all possible ordering that the player can be part of.

SHAP values is a local additive feature (features and predictors are used here synonymously) attribution method ^15^, i.e., it contrastively explains each observation in isolation using a linear function with predicted output as function output and simplified and interpretable feature values as input. Since SHAP values use a linear approximation, for binary classifiers transforming margins using logistic function, SHAP values will be in log-odds space. SHAP values are consistent with respect to feature attribution measurement in tree models, even in the presence of correlated features. If *f* represents the ML model learned (e.g., a trained GBDT model) and *g* represents the local linear explanation model (for a particular observation, $\boldsymbol{x}$), then

$f\left( \boldsymbol{x} \right)=g\left( \boldsymbol{x}^{'} \right)= \emptyset_{0}+ \sum_{i=1}^{M} \emptyset_{i}x_{i}^{'}$,

where, $\emptyset_{0}$ is the output when no input is present, *M* is the number of features, $\boldsymbol{x}^{\boldsymbol{'}}$ is the simplified, dichotomized, and interpretable vector representing ***x*** in the local explanation model and $\emptyset_{i}\mathbb{\in R}$ is the attribution to each feature. According to ^15^, $\emptyset_{i}$ given *f* and ***x*** and satisfying some desirable conditions (local consistency and missingness) is given as ^15^

$$\emptyset_{i}\left( f,\boldsymbol{x} \right)= \sum_{\boldsymbol{z}^{\boldsymbol{'}}\subseteq\boldsymbol{x}^{'}} \frac{\left| \boldsymbol{z}^{'} \right|!\left( M-\left| \boldsymbol{z}^{'} \right|-1 \right)!}{M!} \left[ f_{\boldsymbol{x}}\left( \boldsymbol{z}^{'} \right)- f_{\boldsymbol{x}}\left( \boldsymbol{z}^{'}\backslash i \right) \right],$$

where, $\left| \boldsymbol{z}^{'} \right|$ is the number of features present (non-zero elements) in $\boldsymbol{z}^{\boldsymbol{'}}$. The solution to the above equation is the Shapley values of a conditional expectation function of the original model *f*. We use the recent implementation, called TreeSHAP ^16^ specifically developed for tree-based models, random forest and gradient boosting. TreeSHAP can calculate SHAP values much faster by reducing computational complexity from $\mathcal{O}\left( TL2^{M} \right)$ to $\mathcal{O}\left( TLD^{2} \right)$ (*T* is the number of trees, *L* is the number of leaves, *M* is the number of features, and *D* is the depth of the tree) compared to the previous implementation KernalSHAP ^15^. Also, we observe that TreeSHAP cannot be used with complex ensemble and stacking predictive methods even if such methods provide additional predictive power. We used the Python package ‘SHAP’ version 0.34 for calculating SHAP values.

**Variable selection using SHAP values and correlation**

For each predictor, we calculated variable importance as the mean absolute SHAP value in the training set, as

$\varphi_{i}= \frac{1}{N} \sum_{j=1}^{N} |\emptyset_{ji}|$,

where *N* is the total number of observations in the training set.

We normalized mean absolute SHAP values ($\varphi_{i})$ to 100% and a cut-off value of 0.05% was used to identify ‘important’ predictors. We used Spearman’s ρ (above 0.9) to identify sets of highly correlated predictors and removed all but one (the one recorded for the greatest number of samples) from those sets to produce the final set of important predictors. We plotted SHAP values of important predictors for each sample to understand the direction and magnitude of impact of individual predictors on model output.

**Comparing feature selection using SHAP values and LASSO**

Least absolute shrinkage and selection operator (LASSO) ^17^ is a very popular method for feature selection and has been applied on linear, logistic, probit, Poisson, and Cox regression. LASSO tends to create sparse models and thereby providing reduced number of importance features for further analyses. LASSO uses *L*_1_ penalty to reduce model coefficients of unimportant variables to zero as opposed to nearing zero values in ridge regression. We used scikit-learn^18^ implementation of logistic regression for testing logistic regression with LASSO for feature selection. We imputed missing values for continuous variables with mean values and for ordinal variables with median values. We set the hyper-parameters “penalty” to “l1” and “solver” to “liblinear”. We tried different values for the hyper-parameter *C* (=$\frac{1}{\lambda})$ to see the number of important predictors returned. A smaller value to the hyper-parameter *C* returns fewer number of important variables. For *C* = 0.03, for five random splits, number of features returned had a range of 204-217 and an average of 209 features, similar in number of features selected in our GBDT-SHAP (utilizing CatBoost and SHAP values) models. For GBDT-SHAP models, we took the top 209 features from each run for comparison.

The set of union of features from each run of LASSO was compared to the set of union of features from SHAP values. Overall, LASSO performed well in identifying disease associated features. However, LASSO did not return an association between BMI (or other adiposity indicators), possibly due to a non-linear association with mortality. From within a set of collinear predictors (for example, year of birth and age), LASSO typically picked only a single feature. LASSO did not also select other important predictors such as Townsend deprivation index, or age at cancer diagnosis which were picked up in GBDT-SHAP pipeline. Age at cancer diagnosis is a variable from cancer registration linkage, where information is only included for those participants who had had cancer (84% of the values were missing). While LASSO was not able to detect the relevance of this information for mortality prediction in the presence of missing information, our GBDT-SHAP pipeline was able to rank this feature as the second most important feature.

We then increased the value of *C* to 0.1 to report more than 500 features as important features. We found the feature BMI appearing in positions 186^th^, 241^st^, 264^th^, 488^th^ and not selecting at all as opposed to BMI being selected by GBDT-SHAP always, in positions 52^nd^, 85^th^, 95^th^, 107^th^ and 121^st^, showing relative stability of feature selection by GBDT-SHAP. For Townsend deprivation index, LASSO’s selection was even more distant (positions: 360^th^, 390^th^, 474^th^, not selected, and not selected) as opposed to always being selected by GDBT-SHAP (positions: 61^st^, 69^th^, 82^nd^, 87^th^, and 99^th^) and age at cancer diagnoses not selected at all by LASSO in each of the five runs and GBDT-SHAP consistently selected it as the second most important feature in all the five runs.

**Feature stability**

Feature stability is an important aspect of feature selection for domain experts as it provides assurance to them that the selected features are robust to the perturbation of input data^19^. We compared stability of the features returned by GBDT-SHAP pipeline with features returned by LASSO, and XGBoost with five different built-in feature importance calculation methods, namely, weight, gain, cover, total gain, and total cover.

Similarity between two sets of features *S_i_* and *S_j_* was calculated as ^19^

$S_{s}= \frac{|S_{i} \cap S_{j}|}{|S_{i} \cup S_{j}|}$.

Unadjusted stability measure for *C* runs (resulting in *C* sets of features) was calculated as

$${US}_{C}= \frac{2}{C(C-1)}\sum_{i=1}^{C-1} \sum_{j=i+1}^{C} S_{s}\left( S_{i}, S_{j} \right).$$

We calculated unadjusted feature stability score, ${US}_{C},$ for the top 50, 100, 150, 200, 250 and 300 features for five runs of GBDT-SHAP, LASSO and XGBoost with five different built-in feature importance methods. We found GBDT-SHAP pipeline having consistent higher unadjusted feature stability scores and highest stability scores, from top 200 features onward as shown in Supplementary Fig. S6 online. We observed that LASSO consistently had lower unadjusted feature scores (similar to that of XGBoost’s default feature importance option of ‘weight’) and the difference in the score was highest when top 250 features were selected (0.76 versus 0.58). XGBoost’s ‘gain’ and ‘totalgain’ feature importance methods had higher stability scores for lower number of features, but their feature stability deteriorated steadily for ‘totalgain’ and quickly for ‘gain’ method of feature importance.

One of the issues with the unadjusted stability measure is that it is not adjusted for commonality of a pair of subsets of features obtained purely due to chance, a problem that becomes severe as the number of features in the subset increases. For example, if every feature is included in the subset, then the unadjusted stability score will be the highest for any method and hence not informative. For calculating adjusted stability score, we used the consistency index ($I_{C})$ defined by Kuncheva^20^ as

$$I_{C}\left( S_{i}, S_{j} \right)=\frac{r-\frac{k^{2}}{n}}{k-\frac{k^{2}}{n}}= \frac{rn- k^{2}}{k(n-k)},$$

where, $k=\left| S_{i} \right|=\left| S_{j} \right|, r=\left| S_{i} \cap S_{j} \right|$, *n* is the total number of features and $\frac{k^{2}}{n}$ is the expected cardinality (when features are randomly selected without replacement) of $S_{i} \cap S_{j}.$

Adjusted similarity score was calculated for *C* runs as

$${AS}_{C}= \frac{2}{C(C-1)}\sum_{i=1}^{C-1} \sum_{j=i+1}^{C} I_{C}\left( S_{i}, S_{j} \right).$$

We calculated adjusted feature stability score, ${AS}_{C},$ for the top 50, 100, 150, 200, 250 and 300 features for five runs of GBDT-SHAP, LASSO and XGBoost with five different built-in feature importance methods. We found GBDT-SHAP pipeline having consistent higher adjusted features stability scores and highest stability scores, from top 200 features onward as shown in Supplementary Fig. S7. The patterns of stability scores for all methods were similar to the patterns of unadjusted scores.

# Supplementary Table S1. List of UK Biobank baseline and hospital diagnoses fields considered in the study. There were in total 177 UK Biobank fields (without considering array elements within a field to store multiple values. For example, the field 42104 for recording secondary diagnoses using ICD10 codes had 184 array elements). The baseline fields selected were available for at least 95% of the participants.

| **UK biobank field ID** | **Field description** | **Hierarchical category ^a^** |
| --- | --- | --- |
| **A - Baseline characteristics** | |  |
| 31 | Sex | Population characteristics > Baseline characteristics |
| 34 | Year of birth | Population characteristics > Baseline characteristics |
| 52 | Month of birth | Population characteristics > Baseline characteristics |
| 189 | Townsend deprivation index at recruitment | Population characteristics > Baseline characteristics |
| 21003 | Age when attended assessment center | UK Biobank Assessment Centre > Recruitment > Reception |
| 21022 | Age at recruitment | Population characteristics > Baseline characteristics |
| **B - Sociodemographics** | |  |
| 670 | Type of accommodation lived in | UK Biobank Assessment Centre > Touchscreen > Sociodemographics > Household |
| 680 | Own or rent accommodation lived in | UK Biobank Assessment Centre > Touchscreen > Sociodemographics > Household |
| 699 | Length of time at current address | UK Biobank Assessment Centre > Touchscreen > Sociodemographics > Household |
| 709 | Number in household | UK Biobank Assessment Centre > Touchscreen > Sociodemographics > Household |
| 728 | Number of vehicles in household | UK Biobank Assessment Centre > Touchscreen > Sociodemographics > Household |
| 6138 | Qualifications | UK Biobank Assessment Centre > Touchscreen > Sociodemographics > Education |
| 6139 | Gas or solid-fuel cooking/heating | UK Biobank Assessment Centre > Touchscreen > Sociodemographics > Household |
| 6142 | Current employment status | UK Biobank Assessment Centre > Touchscreen > Sociodemographics > Employment |
| 6146 | Attendance/disability/mobility allowance | UK Biobank Assessment Centre > Touchscreen > Sociodemographics > Other sociodemographic factors |
| 21000 | Ethnic background | UK Biobank Assessment Centre > Touchscreen > Sociodemographics > Ethnicity |
| **C - Lifestyle and environment** | |  |
| 120 | Birth weight known | UK Biobank Assessment Centre > Verbal interview > Early life factors |
| 864 | Number of days/week walked 10+ minutes | UK Biobank Assessment Centre > Touchscreen > Lifestyle and environment > Physical activity |
| 924 | Usual walking pace | UK Biobank Assessment Centre > Touchscreen > Lifestyle and environment > Physical activity |
| 943 | Frequency of stair climbing in last 4 weeks | UK Biobank Assessment Centre > Touchscreen > Lifestyle and environment > Physical activity |
| 1239 | Current tobacco smoking | UK Biobank Assessment Centre > Touchscreen > Lifestyle and environment > Smoking |
| 1289 | Cooked vegetable intake | UK Biobank Assessment Centre > Touchscreen > Lifestyle and environment > Diet |
| 1309 | Fresh fruit intake | UK Biobank Assessment Centre > Touchscreen > Lifestyle and environment > Diet |
| 1329 | Oily fish intake | UK Biobank Assessment Centre > Touchscreen > Lifestyle and environment > Diet |
| 1339 | Non-oily fish intake | UK Biobank Assessment Centre > Touchscreen > Lifestyle and environment > Diet |
| 1349 | Processed meat intake | UK Biobank Assessment Centre > Touchscreen > Lifestyle and environment > Diet |
| 1359 | Poultry intake | UK Biobank Assessment Centre > Touchscreen > Lifestyle and environment > Diet |
| 1369 | Beef intake | UK Biobank Assessment Centre > Touchscreen > Lifestyle and environment > Diet |
| 1379 | Lamb/mutton intake | UK Biobank Assessment Centre > Touchscreen > Lifestyle and environment > Diet |
| 1389 | Pork intake | UK Biobank Assessment Centre > Touchscreen > Lifestyle and environment > Diet |
| 1408 | Cheese intake | UK Biobank Assessment Centre > Touchscreen > Lifestyle and environment > Diet |
| 1418 | Milk type used | UK Biobank Assessment Centre > Touchscreen > Lifestyle and environment > Diet |
| 1428 | Spread type | UK Biobank Assessment Centre > Touchscreen > Lifestyle and environment > Diet |
| 1438 | Bread intake | UK Biobank Assessment Centre > Touchscreen > Lifestyle and environment > Diet |
| 1448 | Bread type | UK Biobank Assessment Centre > Touchscreen > Lifestyle and environment > Diet |
| 1478 | Salt added to food | UK Biobank Assessment Centre > Touchscreen > Lifestyle and environment > Diet |
| 1488 | Tea intake | UK Biobank Assessment Centre > Touchscreen > Lifestyle and environment > Diet |
| 1518 | Hot drink temperature | UK Biobank Assessment Centre > Touchscreen > Lifestyle and environment > Diet |
| 1538 | Major dietary changes in the last 5 years | UK Biobank Assessment Centre > Touchscreen > Lifestyle and environment > Diet |
| 1548 | Variation in diet | UK Biobank Assessment Centre > Touchscreen > Lifestyle and environment > Diet |
| 1647 | Country of birth (UK/elsewhere) | UK Biobank Assessment Centre > Touchscreen > Early life factors |
| 1687 | Comparative body size at age 10 | UK Biobank Assessment Centre > Touchscreen > Early life factors |
| 1697 | Comparative height size at age 10 | UK Biobank Assessment Centre > Touchscreen > Early life factors |
| 1707 | Handedness (chirality/laterality) | UK Biobank Assessment Centre > Touchscreen > Early life factors |
| 1717 | Skin color | UK Biobank Assessment Centre > Touchscreen > Lifestyle and environment > Sun exposure |
| 1727 | Ease of skin tanning | UK Biobank Assessment Centre > Touchscreen > Lifestyle and environment > Sun exposure |
| 1747 | Hair color (natural, before greying) | UK Biobank Assessment Centre > Touchscreen > Lifestyle and environment > Sun exposure |
| 1767 | Adopted as a child | UK Biobank Assessment Centre > Touchscreen > Early life factors |
| 1777 | Part of a multiple birth | UK Biobank Assessment Centre > Touchscreen > Early life factors |
| 2267 | Use of sun/UV protection | UK Biobank Assessment Centre > Touchscreen > Lifestyle and environment > Sun exposure |
| 6144 | Never eat eggs, dairy, wheat, sugar | UK Biobank Assessment Centre > Touchscreen > Lifestyle and environment > Diet |
| 6162 | Types of transport used (excluding work) | UK Biobank Assessment Centre > Touchscreen > Lifestyle and environment > Physical activity |
| 6164 | Types of physical activity in last 4 weeks | UK Biobank Assessment Centre > Touchscreen > Lifestyle and environment > Physical activity |
| 20116 | Smoking status | UK Biobank Assessment Centre > Touchscreen > Lifestyle and environment > Smoking |
| 20160 | Ever smoked | UK Biobank Assessment Centre > Touchscreen > Lifestyle and environment > Smoking |
| **D - Physical measurements** | |  |
| 21 | Weight method | UK Biobank Assessment Centre > Physical measures > Anthropometry > Body size measures |
| 46 | Hand grip strength (left) | UK Biobank Assessment Centre > Physical measures > Hand grip strength |
| 47 | Hand grip strength (right) | UK Biobank Assessment Centre > Physical measures > Hand grip strength |
| 48 | Waist circumference | UK Biobank Assessment Centre > Physical measures > Anthropometry > Body size measures |
| 49 | Hip circumference | UK Biobank Assessment Centre > Physical measures > Anthropometry > Body size measures |
| 50 | Standing height | UK Biobank Assessment Centre > Physical measures > Anthropometry > Body size measures |
| 3088 | Contra-indications for spirometry | UK Biobank Assessment Centre > Physical measures > Spirometry |
| 20015 | Sitting height | UK Biobank Assessment Centre > Physical measures > Anthropometry > Body size measures |
| 21001 | Body mass index (BMI) | UK Biobank Assessment Centre > Physical measures > Anthropometry > Body size measures |
| 21002 | Weight | UK Biobank Assessment Centre > Physical measures > Anthropometry > Body size measures |
| 23098 | Weight | UK Biobank Assessment Centre > Physical measures > Anthropometry > Impedance measures |
| 23099 | Body fat percentage | UK Biobank Assessment Centre > Physical measures > Anthropometry > Impedance measures |
| 23100 | Whole body fat mass | UK Biobank Assessment Centre > Physical measures > Anthropometry > Impedance measures |
| 23101 | Whole body fat-free mass | UK Biobank Assessment Centre > Physical measures > Anthropometry > Impedance measures |
| 23102 | Whole body water mass | UK Biobank Assessment Centre > Physical measures > Anthropometry > Impedance measures |
| 23104 | Body mass index (BMI) | UK Biobank Assessment Centre > Physical measures > Anthropometry > Impedance measures |
| 23105 | Basal metabolic rate | UK Biobank Assessment Centre > Physical measures > Anthropometry > Impedance measures |
| 23106 | Impedance of whole body | UK Biobank Assessment Centre > Physical measures > Anthropometry > Impedance measures |
| 23107 | Impedance of leg (right) | UK Biobank Assessment Centre > Physical measures > Anthropometry > Impedance measures |
| 23108 | Impedance of leg (left) | UK Biobank Assessment Centre > Physical measures > Anthropometry > Impedance measures |
| 23109 | Impedance of arm (right) | UK Biobank Assessment Centre > Physical measures > Anthropometry > Impedance measures |
| 23110 | Impedance of arm (left) | UK Biobank Assessment Centre > Physical measures > Anthropometry > Impedance measures |
| 23111 | Leg fat percentage (right) | UK Biobank Assessment Centre > Physical measures > Anthropometry > Impedance measures |
| 23112 | Leg fat mass (right) | UK Biobank Assessment Centre > Physical measures > Anthropometry > Impedance measures |
| 23113 | Leg fat-free mass (right) | UK Biobank Assessment Centre > Physical measures > Anthropometry > Impedance measures |
| 23114 | Leg predicted mass (right) | UK Biobank Assessment Centre > Physical measures > Anthropometry > Impedance measures |
| 23115 | Leg fat percentage (left) | UK Biobank Assessment Centre > Physical measures > Anthropometry > Impedance measures |
| 23116 | Leg fat mass (left) | UK Biobank Assessment Centre > Physical measures > Anthropometry > Impedance measures |
| 23117 | Leg fat-free mass (left) | UK Biobank Assessment Centre > Physical measures > Anthropometry > Impedance measures |
| 23118 | Leg predicted mass (left) | UK Biobank Assessment Centre > Physical measures > Anthropometry > Impedance measures |
| 23119 | Arm fat percentage (right) | UK Biobank Assessment Centre > Physical measures > Anthropometry > Impedance measures |
| 23120 | Arm fat mass (right) | UK Biobank Assessment Centre > Physical measures > Anthropometry > Impedance measures |
| 23121 | Arm fat-free mass (right) | UK Biobank Assessment Centre > Physical measures > Anthropometry > Impedance measures |
| 23122 | Arm predicted mass (right) | UK Biobank Assessment Centre > Physical measures > Anthropometry > Impedance measures |
| 23123 | Arm fat percentage (left) | UK Biobank Assessment Centre > Physical measures > Anthropometry > Impedance measures |
| 23124 | Arm fat mass (left) | UK Biobank Assessment Centre > Physical measures > Anthropometry > Impedance measures |
| 23125 | Arm fat-free mass (left) | UK Biobank Assessment Centre > Physical measures > Anthropometry > Impedance measures |
| 23126 | Arm predicted mass (left) | UK Biobank Assessment Centre > Physical measures > Anthropometry > Impedance measures |
| 23127 | Trunk fat percentage | UK Biobank Assessment Centre > Physical measures > Anthropometry > Impedance measures |
| 23128 | Trunk fat mass | UK Biobank Assessment Centre > Physical measures > Anthropometry > Impedance measures |
| 23129 | Trunk fat-free mass | UK Biobank Assessment Centre > Physical measures > Anthropometry > Impedance measures |
| 23130 | Trunk predicted mass | UK Biobank Assessment Centre > Physical measures > Anthropometry > Impedance measures |
| **E - Cognitive function** | |  |
| 398 | Number of correct matches in round | UK Biobank Assessment Centre > Cognitive function > Pairs matching |
| 399 | Number of incorrect matches in round | UK Biobank Assessment Centre > Cognitive function > Pairs matching |
| 400 | Time to complete round | UK Biobank Assessment Centre > Cognitive function > Pairs matching |
| 403 | Number of times snap-button pressed | UK Biobank Assessment Centre > Cognitive function > Reaction time |
| 404 | Duration to first press of snap-button in each round | UK Biobank Assessment Centre > Cognitive function > Reaction time |
| 20023 | Mean time to correctly identify matches | UK Biobank Assessment Centre > Cognitive function > Reaction time |
| **F - Psychosocial factors** | |  |
| 1920 | Mood swings | UK Biobank Assessment Centre > Touchscreen > Psychosocial factors > Mental health |
| 1930 | Miserableness | UK Biobank Assessment Centre > Touchscreen > Psychosocial factors > Mental health |
| 1940 | Irritability | UK Biobank Assessment Centre > Touchscreen > Psychosocial factors > Mental health |
| 1950 | Sensitivity / hurt feelings | UK Biobank Assessment Centre > Touchscreen > Psychosocial factors > Mental health |
| 1960 | Fed-up feelings | UK Biobank Assessment Centre > Touchscreen > Psychosocial factors > Mental health |
| 1970 | Nervous feelings | UK Biobank Assessment Centre > Touchscreen > Psychosocial factors > Mental health |
| 1980 | Worrier / anxious feelings | UK Biobank Assessment Centre > Touchscreen > Psychosocial factors > Mental health |
| 1990 | Tense / 'highly strung' | UK Biobank Assessment Centre > Touchscreen > Psychosocial factors > Mental health |
| 2000 | Worry too long after embarrassment | UK Biobank Assessment Centre > Touchscreen > Psychosocial factors > Mental health |
| 2010 | Suffer from 'nerves' | UK Biobank Assessment Centre > Touchscreen > Psychosocial factors > Mental health |
| 2020 | Loneliness, isolation | UK Biobank Assessment Centre > Touchscreen > Psychosocial factors > Mental health |
| 2030 | Guilty feelings | UK Biobank Assessment Centre > Touchscreen > Psychosocial factors > Mental health |
| 2040 | Risk taking | UK Biobank Assessment Centre > Touchscreen > Psychosocial factors > Mental health |
| 2050 | Frequency of depressed mood in last 2 weeks | UK Biobank Assessment Centre > Touchscreen > Psychosocial factors > Mental health |
| 2060 | Frequency of unenthusiasm / disinterest in last 2 weeks | UK Biobank Assessment Centre > Touchscreen > Psychosocial factors > Mental health |
| 2070 | Frequency of tenseness / restlessness in last 2 weeks | UK Biobank Assessment Centre > Touchscreen > Psychosocial factors > Mental health |
| 2080 | Frequency of tiredness / lethargy in last 2 weeks | UK Biobank Assessment Centre > Touchscreen > Psychosocial factors > Mental health |
| 2090 | Seen doctor (GP) for nerves, anxiety, tension, or depression | UK Biobank Assessment Centre > Touchscreen > Psychosocial factors > Mental health |
| 2100 | Seen a psychiatrist for nerves, anxiety, tension, or depression | UK Biobank Assessment Centre > Touchscreen > Psychosocial factors > Mental health |
| 6145 | Illness, injury, bereavement, stress in last 2 years | UK Biobank Assessment Centre > Touchscreen > Psychosocial factors > Mental health |
| **G - Self-reported diseases** | |  |
| 134 | Number of self-reported cancers | UK Biobank Assessment Centre > Verbal interview > Medical conditions |
| 135 | Number of self-reported non-cancer illnesses | UK Biobank Assessment Centre > Verbal interview > Medical conditions |
| **H - Medications & Operations** | |  |
| 136 | Number of operations, self-reported | UK Biobank Assessment Centre > Verbal interview > Operations |
| 137 | Number of treatments/medications taken | UK Biobank Assessment Centre > Verbal interview > Medications |
| 3079 | Pacemaker | UK Biobank Assessment Centre > Verbal interview > Operations |
| **I - Health and medical history** | |  |
| 1797 | Father still alive | UK Biobank Assessment Centre > Touchscreen > Family history |
| 1835 | Mother still alive | UK Biobank Assessment Centre > Touchscreen > Family history |
| 1873 | Number of full brothers | UK Biobank Assessment Centre > Touchscreen > Family history |
| 1883 | Number of full sisters | UK Biobank Assessment Centre > Touchscreen > Family history |
| 2178 | Overall health rating | UK Biobank Assessment Centre > Touchscreen > Health and medical history > General health |
| 2188 | Long-standing illness, disability, or infirmity | UK Biobank Assessment Centre > Touchscreen > Health and medical history > General health |
| 2207 | Wears glasses or contact lenses | UK Biobank Assessment Centre > Touchscreen > Health and medical history > Eyesight |
| 2227 | Other eye problems | UK Biobank Assessment Centre > Touchscreen > Health and medical history > Eyesight |
| 2247 | Hearing difficulty/problems | UK Biobank Assessment Centre > Touchscreen > Health and medical history > Hearing |
| 2257 | Hearing difficulty/problems with background noise | UK Biobank Assessment Centre > Touchscreen > Health and medical history > Hearing |
| 2296 | Falls in the last year | UK Biobank Assessment Centre > Touchscreen > Health and medical history > General health |
| 2306 | Weight change compared with 1 year ago | UK Biobank Assessment Centre > Touchscreen > Health and medical history > General health |
| 2316 | Wheeze or whistling in the chest in last year | UK Biobank Assessment Centre > Touchscreen > Health and medical history > Breathing |
| 2335 | Chest pain or discomfort | UK Biobank Assessment Centre > Touchscreen > Health and medical history > Chest pain |
| 2345 | Ever had bowel cancer screening | UK Biobank Assessment Centre > Touchscreen > Health and medical history > Cancer screening |
| 2443 | Diabetes diagnosed by doctor | UK Biobank Assessment Centre > Touchscreen > Health and medical history > Medical conditions |
| 2453 | Cancer diagnosed by doctor | UK Biobank Assessment Centre > Touchscreen > Health and medical history > Medical conditions |
| 2463 | Fractured/broken bones in last 5 years | UK Biobank Assessment Centre > Touchscreen > Health and medical history > Medical conditions |
| 2473 | Other serious medical condition/disability diagnosed by doctor | UK Biobank Assessment Centre > Touchscreen > Health and medical history > Medical conditions |
| 2492 | Taking other prescription medications | UK Biobank Assessment Centre > Touchscreen > Health and medical history > Medication |
| 6149 | Mouth/teeth dental problems | UK Biobank Assessment Centre > Touchscreen > Health and medical history > Mouth |
| 6150 | Vascular/heart problems diagnosed by doctor | UK Biobank Assessment Centre > Touchscreen > Health and medical history > Medical conditions |
| 6152 | Blood clot, DVT, bronchitis, emphysema, asthma, rhinitis, eczema, allergy diagnosed by doctor | UK Biobank Assessment Centre > Touchscreen > Health and medical history > Medical conditions |
| 6154 | Medication for pain relief, constipation, heartburn | UK Biobank Assessment Centre > Touchscreen > Health and medical history > Medication |
| 6155 | Vitamin and mineral supplements | UK Biobank Assessment Centre > Touchscreen > Health and medical history > Medication |
| 6159 | Pain type(s) experienced in last month | UK Biobank Assessment Centre > Touchscreen > Health and medical history > Pain |
| 6179 | Mineral and other dietary supplements | UK Biobank Assessment Centre > Touchscreen > Health and medical history > Medication |
| **J - Hospital diagnoses** | |  |
| 40006 | Type of cancer: ICD10 | Health-related outcomes > Cancer register |
| 40008 | Age at cancer diagnosis | Health-related outcomes > Cancer register |
| 40009 | Reported occurrences of cancer | Health-related outcomes > Cancer register |
| 40011 | Histology of cancer tumor | Health-related outcomes > Cancer register |
| 40012 | Behavior of cancer tumor | Health-related outcomes > Cancer register |
| 40013 | Type of cancer: ICD9 | Health-related outcomes > Cancer register |
| 40019 | Cancer report format | Health-related outcomes > Cancer register |
| 41201 | External causes - ICD10 | Health-related outcomes > Hospital inpatient > Summary Diagnoses |
| 41202 | Diagnoses - main ICD10 | Health-related outcomes > Hospital inpatient > Summary Diagnoses |
| 41203 | Diagnoses - main ICD9 | Health-related outcomes > Hospital inpatient > Summary Diagnoses |
| 41204 | Diagnoses - secondary ICD10 | Health-related outcomes > Hospital inpatient > Summary Diagnoses |
| 41205 | Diagnoses - secondary ICD9 | Health-related outcomes > Hospital inpatient > Summary Diagnoses |
| 41219 | Anesthetics administered during delivery | Health-related outcomes > Hospital inpatient > Summary Maternity |
| 41220 | Anesthetics administered post delivery | Health-related outcomes > Hospital inpatient > Summary Maternity |
| 41221 | Delivery methods | Health-related outcomes > Hospital inpatient > Summary Maternity |
| 41222 | Delivery onset methods | Health-related outcomes > Hospital inpatient > Summary Maternity |
| 41223 | Delivery places | Health-related outcomes > Hospital inpatient > Summary Maternity |
| 41224 | Intended delivery places | Health-related outcomes > Hospital inpatient > Summary Maternity |
| 41225 | Resuscitation methods | Health-related outcomes > Hospital inpatient > Summary Maternity |
| 41226 | Sex of baby | Health-related outcomes > Hospital inpatient > Summary Maternity |
| 41227 | Status of baby at birth | Health-related outcomes > Hospital inpatient > Summary Maternity |
| 41228 | Statuses of person conducting delivery | Health-related outcomes > Hospital inpatient > Summary Maternity |

^a^ More information on UK biobank field categories can be found at the UK biobank website (<https://biobank.ndph.ox.ac.uk/showcase/cats.cgi>)

**Supplementary Table S2.** Category wise count of predictors before and after pre-processing using PHESANT (PHEnome Scan Analysis) software package. Important predictors are the predictors identified using SHAP (SHapley Additive exPlanation) values passing the selected SHAP value threshold of 0.05% and after further elimination of predictors using Spearman’s ρ.

| **Category** | **All predictors** ^a^ | | **Important predictors** ^b^ | |
| --- | --- | --- | --- | --- |
|  | **UK Biobank fields** | **Derived predictors** ^c^ | **UK Biobank fields** | **Derived predictors** ^c^ |
| A - Baseline characteristics | 6 | 6 | 4 | 4 |
| B - Sociodemographics | 10 | 29 | 9 | 15 |
| C - Lifestyle and environment | 39 | 51 | 25 | 28 |
| D - Physical measurements | 42 | 42 | 13 | 13 |
| E - Cognitive function | 6 | 6 | 3 | 3 |
| F - Psychosocial factors | 20 | 26 | 8 | 10 |
| G - Self-reported diseases | 2 | 2 | 2 | 2 |
| H - Medications & Operations | 3 | 3 | 2 | 2 |
| I - Health and medical history | 27 | 69 | 21 | 31 |
| J - Hospital diagnoses | 22 | 11,405 | 8 | 85 |
| **Total** | **177** | **11,639** | **95** | **193** |

^a^ Fields considered in the gradient boosting decision tree (GBDT) models with all predictors in its input.

^b^ Fields considered in the GBDT models with only important predictors in its input.

^c^ Derived predictors are the output predictors of PHESANT pre-processing and were used as input to GBDT models.

**Supplementary Table S3.** Category wise listing of important predictors identified. Three sets of SHAP values are provided: a) SHAP values (normalized for 100%) from a gradient boosting decision trees (GBDT) model with all predictors in its input totaling 84%, b) SHAP values adjusted for a total of 100% and c) SHAP values from a GBDT model with only important predictors in its input.

| **Predictor** ^a^ | **SHAP value**  **(all)**^b^ | **SHAP value**  **(all adj)** ^c^ | **SHAP value**  **(im.)** ^d^ |
| --- | --- | --- | --- |
| **A - Baseline characteristics** |  |  |  |
| Age at recruitment | 0.46 | 0.55 | 4.51 |
| Sex—male | 3.02 | 3.59 | 3.84 |
| Townsend deprivation index | 0.20 | 0.24 | 0.26 |
| Month of birth | 0.30 | 0.36 | 0.23 |
| **A - Baseline characteristics Total** | **3.98** | **4.74** | **8.85** |
| **B - Sociodemographics** |  |  |  |
| In paid employment or self-employed | 2.64 | 3.15 | 2.89 |
| Number in household | 1.17 | 1.39 | 1.17 |
| Number of vehicles in household | 1.15 | 1.37 | 1.08 |
| Length of time at current address | 0.67 | 0.80 | 0.78 |
| Ethnic background | 0.57 | 0.68 | 0.78 |
| Attendance/disability/mobility allowance—none | 0.75 | 0.89 | 0.74 |
| Attendance/disability/mobility allowance - disability | 0.25 | 0.29 | 0.21 |
| Gas or solid-fuel cooking/heating - a gas fire that you use regularly in winter time | 0.14 | 0.17 | 0.19 |
| Gas or solid-fuel cooking/heating - an open solid fuel fire that you use regularly in winter time | 0.10 | 0.12 | 0.19 |
| Current employment status - unable to work because of sickness or disability | 0.24 | 0.29 | 0.18 |
| Qualifications - CSEs or equivalent | 0.08 | 0.09 | 0.16 |
| Qualifications - A levels/AS levels or equivalent | 0.07 | 0.09 | 0.13 |
| Type of accommodation lived in—house, flat, mobile, sheltered, care home | 0.05 | 0.06 | 0.13 |
| Gas or solid-fuel cooking/heating—none | 0.08 | 0.09 | 0.12 |
| Current employment status - unemployed | 0.07 | 0.08 | 0.03 |
| **B - Sociodemographics Total** | **8.03** | **9.56** | **8.80** |
| **C - Lifestyle and environment** |  |  |  |
| Current tobacco smoking—more frequently | 1.80 | 2.14 | 1.82 |
| Smoking status—never, previous, current | 1.41 | 1.67 | 1.38 |
| Usual walking pace | 1.06 | 1.27 | 1.06 |
| Physical activity in last 4 weeks - other exercises (e.g.: swimming, cycling, keep fit, bowling) | 0.78 | 0.92 | 0.68 |
| Use of sun/UV protection—always | 0.62 | 0.74 | 0.67 |
| Frequency of stair climbing in last 4 weeks | 0.45 | 0.54 | 0.51 |
| Poultry intake | 0.30 | 0.36 | 0.40 |
| Tea intake | 0.35 | 0.42 | 0.39 |
| Processed meat intake | 0.27 | 0.32 | 0.33 |
| Ever smoked | 0.24 | 0.28 | 0.29 |
| Beef intake | 0.25 | 0.30 | 0.26 |
| Light DIY physical activity in last 4 weeks | 0.20 | 0.24 | 0.24 |
| Cooked vegetable intake | 0.27 | 0.32 | 0.22 |
| Bread intake | 0.11 | 0.13 | 0.22 |
| Eat eggs, diary, wheat, sugar2 | 0.12 | 0.14 | 0.21 |
| Bread type | 0.19 | 0.23 | 0.21 |
| Comparative height size at age 10 | 0.16 | 0.19 | 0.21 |
| Types of physical activity in last 4 weeks - Heavy DIY (e.g.: weeding, lawn mowing, carpentry, digging) | 0.16 | 0.19 | 0.20 |
| Pork intake | 0.14 | 0.16 | 0.19 |
| Number of days/week walked 10+ minutes | 0.13 | 0.15 | 0.17 |
| Skin color—less fair | 0.11 | 0.14 | 0.16 |
| Types of physical activity in last 4 weeks - strenuous sports | 0.05 | 0.06 | 0.15 |
| Salt added to food | 0.10 | 0.12 | 0.15 |
| Cheese intake | 0.07 | 0.09 | 0.14 |
| Country of birth (UK/elsewhere) | 0.13 | 0.16 | 0.13 |
| Handedness—right-handed, left-handed, use both hands | 0.07 | 0.09 | 0.12 |
| Hot drink temperature—less hot | 0.08 | 0.09 | 0.11 |
| Milk type used | 0.07 | 0.08 | 0.10 |
| **C - Lifestyle and environment Total** | **9.70** | **11.54** | **10.72** |
| **D - Physical measurements** |  |  |  |
| Leg fat percentage (right) | 0.68 | 0.81 | 1.79 |
| Waist circumference | 1.24 | 1.47 | 1.44 |
| Impedance of leg (right) | 0.22 | 0.26 | 0.60 |
| Sitting height | 0.43 | 0.51 | 0.54 |
| Body mass index (BMI) | 0.30 | 0.35 | 0.51 |
| Hand grip strength (right) | 0.11 | 0.14 | 0.33 |
| Whole body fat-free mass | 0.11 | 0.13 | 0.25 |
| Impedance of arm (left) | 0.13 | 0.15 | 0.21 |
| Arm fat mass (right) | 0.07 | 0.08 | 0.20 |
| Standing height | 0.13 | 0.16 | 0.18 |
| Hip circumference | 0.10 | 0.12 | 0.18 |
| Weight | 0.06 | 0.07 | 0.16 |
| Contra-indications for spirometry | 0.13 | 0.15 | 0.13 |
| **D - Physical measurements Total** | **3.71** | **4.41** | **6.51** |
| **E - Cognitive function** |  |  |  |
| Mean time to correctly identify matches | 0.57 | 0.68 | 0.96 |
| Time to complete round | 0.55 | 0.65 | 0.63 |
| Number of incorrect matches in round | 0.09 | 0.10 | 0.17 |
| **E - Cognitive function Total** | **1.21** | **1.44** | **1.76** |
| **F - Psychosocial factors** |  |  |  |
| Worry too long after embarrassment | 0.55 | 0.65 | 0.58 |
| Illness, injury, bereavement, stress in last 2 years - Serious illness, injury, or assault of a close relative | 0.20 | 0.24 | 0.30 |
| Risk taking | 0.15 | 0.17 | 0.25 |
| Fed-up feelings | 0.11 | 0.13 | 0.19 |
| Sensitivity / hurt feelings | 0.17 | 0.20 | 0.18 |
| Guilty feelings | 0.08 | 0.09 | 0.17 |
| Irritability | 0.06 | 0.07 | 0.16 |
| Illness, injury, bereavement, stress in last 2 years - serious illness, injury, or assault to yourself | 0.08 | 0.10 | 0.13 |
| Worrier / anxious feelings | 0.09 | 0.11 | 0.09 |
| Illness, injury, bereavement, stress in last 2 years - Death of a close relative | 0.07 | 0.08 | 0.08 |
| **F - Psychosocial factors Total** | **1.56** | **1.85** | **2.14** |
| **G - Self-reported diseases** |  |  |  |
| Number of self-reported cancers | 0.61 | 0.73 | 0.77 |
| Number of self-reported non-cancer illnesses | 0.17 | 0.20 | 0.20 |
| **G - Self-reported diseases Total** | **0.78** | **0.93** | **0.97** |
| **H - Medications & Operations** |  |  |  |
| Number of treatments/medications taken | 0.28 | 0.33 | 0.36 |
| Number of operations, self-reported | 0.05 | 0.06 | 0.06 |
| **H - Medications & Operations Total** | **0.33** | **0.39** | **0.42** |
| **I - Health and medical history** |  |  |  |
| Long-standing illness, disability, or infirmity | 1.51 | 1.80 | 1.44 |
| Overall health rating—poorer | 1.51 | 1.80 | 1.34 |
| Ever had bowel cancer screening | 0.93 | 1.10 | 1.03 |
| Diabetes diagnosed by doctor | 0.49 | 0.59 | 0.50 |
| Vascular/heart problems diagnosed by doctor - heart attack | 0.43 | 0.52 | 0.47 |
| Mother still alive | 0.24 | 0.28 | 0.43 |
| Medication for pain relief, constipation, heartburn - paracetamol | 0.32 | 0.39 | 0.37 |
| Mouth/teeth dental problems - dentures | 0.37 | 0.44 | 0.37 |
| Hearing difficulty/problems with background noise | 0.46 | 0.54 | 0.34 |
| Number of full brothers | 0.27 | 0.33 | 0.29 |
| Pain type(s) experienced in last month - headache | 0.17 | 0.20 | 0.26 |
| Pain type(s) experienced in last month - knee pain | 0.14 | 0.17 | 0.25 |
| Mineral and other dietary supplements - glucosamine | 0.20 | 0.24 | 0.24 |
| Medication for pain relief, constipation, heartburn—none | 0.13 | 0.15 | 0.24 |
| No vascular/heart problems diagnosed by doctor | 0.11 | 0.13 | 0.22 |
| Taking other prescription medications | 0.18 | 0.22 | 0.22 |
| Number of full sisters | 0.10 | 0.12 | 0.21 |
| Current employment status - doing unpaid or voluntary work | 0.17 | 0.20 | 0.20 |
| Father still alive | 0.09 | 0.11 | 0.20 |
| Fractured/broken bones in last 5 years | 0.13 | 0.15 | 0.19 |
| Other serious medical condition/disability diagnosed by doctor | 0.11 | 0.13 | 0.18 |
| Wheeze or whistling in the chest in last year | 0.24 | 0.29 | 0.18 |
| Pain type(s) experienced in last month—none | 0.21 | 0.25 | 0.17 |
| Pain type(s) experienced in last month - neck or shoulder pain | 0.10 | 0.11 | 0.16 |
| Hay fever, allergic rhinitis or eczema diagnosed by doctor | 0.08 | 0.09 | 0.14 |
| Weight change compared with 1 year ago—lost, same, gained | 0.09 | 0.11 | 0.13 |
| Medication for pain relief, constipation, heartburn - ibuprofen (e.g. Nurofen) | 0.06 | 0.07 | 0.12 |
| Hearing difficulty/problems | 0.06 | 0.07 | 0.12 |
| Mouth/teeth dental problems—none | 0.07 | 0.08 | 0.08 |
| Mineral and other dietary supplements—none | 0.10 | 0.11 | 0.07 |
| Mineral and other dietary supplements - fish oil (including cod liver oil) | 0.05 | 0.06 | 0.06 |
| **I - Health and medical history Total** | **9.12** | **10.85** | **10.22** |
| **J - Hospital diagnoses** |  |  |  |
| Sec. - 251.5 palliative care | 8.86 | 10.55 | 9.33 |
| Age at cancer diagnosis | 5.86 | 6.97 | 5.91 |
| Malignant, primary site cancer tumor | 4.29 | 5.10 | 4.23 |
| Sec. - Z51.1 chemotherapy session for neoplasm | 4.29 | 5.10 | 4.20 |
| Sec. - C78.7 secondary malignant neoplasm of liver | 2.71 | 3.23 | 2.87 |
| Sec. - C79.5 Sec. malignant neoplasm of bone and bone marrow | 1.62 | 1.93 | 2.08 |
| Sec. - N17.9 acute renal failure, unspecified | 1.47 | 1.75 | 1.45 |
| Sec. - I10 essential (primary) hypertension | 0.98 | 1.17 | 1.00 |
| Sec. - I48 atrial fibrillation and flutter | 0.87 | 1.04 | 0.85 |
| Histology of cancer tumor - Basal cell carcinoma, NOS | 0.80 | 0.96 | 0.84 |
| Sec. - J90 pleural effusion, not elsewhere classified | 0.84 | 0.99 | 0.80 |
| Reported occurrences of cancer | 0.67 | 0.80 | 0.79 |
| Sec. - Z86.4 personal history of psychoactive substance abuse | 0.77 | 0.92 | 0.74 |
| Sec. - C78.0 secondary malignant neoplasm of lung | 0.46 | 0.55 | 0.65 |
| C61 malignant neoplasm of prostate | 0.41 | 0.48 | 0.56 |
| Sec. - Z51.8 other specified medical care | 0.42 | 0.50 | 0.51 |
| Sec. - I46.9 cardiac arrest, unspecified | 0.42 | 0.50 | 0.45 |
| Sec. - R18 ascites | 0.33 | 0.39 | 0.40 |
| Sec. - J96.9 respiratory failure, unspecified | 0.36 | 0.43 | 0.40 |
| Sec. - C78.6 Sec. malignant neoplasm of retroperitoneum and peritoneum | 0.41 | 0.49 | 0.39 |
| Main - C34.9 bronchus or lung, unspecified | 0.31 | 0.36 | 0.36 |
| Histology of cancer tumor - adenocarcinoma-NOS | 0.17 | 0.20 | 0.35 |
| Behavior of cancer tumor - carcinoma in situ cancer tumor | 0.30 | 0.35 | 0.35 |
| Sec. - N39.0 urinary tract infection, site not specified | 0.35 | 0.41 | 0.33 |
| Sec. - E87.2 acidosis | 0.26 | 0.31 | 0.32 |
| Sec. - J22 unspecified acute lower respiratory infection | 0.26 | 0.31 | 0.30 |
| Main - Z08.0 follow-up exam. after surgery for malignant neoplasm | 0.23 | 0.27 | 0.29 |
| Sec. - F10.2 dependence syndrome | 0.24 | 0.29 | 0.29 |
| Main - C50.9 breast, unspecified | 0.13 | 0.15 | 0.29 |
| Sec. - J18.1 lobar pneumonia, unspecified | 0.26 | 0.31 | 0.28 |
| Main - M17.9 gonarthrosis, unspecified | 0.24 | 0.29 | 0.28 |
| Sec. - J84.1 other interstitial pulmonary diseases with fibrosis | 0.24 | 0.28 | 0.28 |
| Sec. - I95.9 hypotension, unspecified | 0.20 | 0.23 | 0.28 |
| Histology of cancer tumor - duct adenocarcinoma | 0.21 | 0.24 | 0.27 |
| Histology of cancer tumor - glioblastoma-NOS | 0.23 | 0.27 | 0.26 |
| Sec. - R56.8 other and unspecified convulsions | 0.18 | 0.21 | 0.24 |
| Main - G12.2 motor neuron disease | 0.21 | 0.25 | 0.23 |
| Sec. - D64.9 anemia, unspecified | 0.21 | 0.25 | 0.22 |
| Cancer report format | 0.34 | 0.40 | 0.22 |
| Sec. - R63.4 abnormal weight loss | 0.18 | 0.22 | 0.21 |
| Sec. - F10.1 harmful use | 0.18 | 0.21 | 0.20 |
| Sec. - I50.0 congestive heart failure | 0.14 | 0.17 | 0.20 |
| Main - K63.5 polyp of colon | 0.11 | 0.14 | 0.20 |
| Sec. - F32.9 depressive episode, unspecified | 0.07 | 0.09 | 0.19 |
| Main - K21.9 gastro-esophageal reflux disease without esophagitis | 0.12 | 0.14 | 0.19 |
| Sec. - A41.9 septicemia, unspecified | 0.13 | 0.15 | 0.18 |
| Sec. - I50.1 left ventricular failure | 0.16 | 0.19 | 0.18 |
| Main - J18.1 lobar pneumonia, unspecified | 0.17 | 0.21 | 0.18 |
| Sec. - G20 Parkinson's disease | 0.12 | 0.14 | 0.17 |
| Main - R69 unknown and unspecified causes of morbidity | 0.10 | 0.12 | 0.16 |
| Sec. - M17.9 gonarthrosis, unspecified | 0.08 | 0.09 | 0.16 |
| Sec. - I46.0 cardiac arrest with successful resuscitation | 0.15 | 0.18 | 0.16 |
| Main - J90 pleural effusion, not elsewhere classified | 0.09 | 0.11 | 0.15 |
| Main - M16.9 coxarthrosis, unspecified | 0.09 | 0.10 | 0.15 |
| Sec. - F03 unspecified dementia | 0.11 | 0.13 | 0.14 |
| Main - C79.3 Sec. malignant neoplasm of brain and cerebral meninges | 0.09 | 0.10 | 0.13 |
| Main - D64.9 anemia, unspecified | 0.09 | 0.10 | 0.13 |
| Sec. - J69.0 pneumonitis due to food and vomit | 0.09 | 0.11 | 0.13 |
| Histology of cancer tumor - neoplasm | 0.08 | 0.09 | 0.13 |
| Sec. - Z51.3 blood transfusion without reported diagnosis | 0.09 | 0.11 | 0.12 |
| Histology of cancer tumor - carcinoma-NOS | 0.08 | 0.10 | 0.12 |
| Sec. - R41.0 disorientation, unspecified | 0.07 | 0.08 | 0.11 |
| Main - K40.9 unilateral or unspecified inguinal hernia | 0.07 | 0.09 | 0.11 |
| Sec. - R94.5 abnormal results of liver function studies | 0.08 | 0.09 | 0.11 |
| Sec. - E87.1 hypo-osmolality and hyponatremia | 0.07 | 0.09 | 0.11 |
| Main - I61.9 intracerebral hemorrhage, unspecified | 0.11 | 0.13 | 0.10 |
| Main - I63.9 cerebral infarction, unspecified | 0.08 | 0.10 | 0.10 |
| Sec. - Z86.7 personal history of diseases of the circulatory system | 0.11 | 0.13 | 0.10 |
| Sec. - J18.9 pneumonia, unspecified | 0.05 | 0.07 | 0.10 |
| Main - J22 unspecified acute lower respiratory infection | 0.07 | 0.09 | 0.10 |
| Main - C44.3 Other/unspecified skin of other and unspecified parts of face | 0.05 | 0.06 | 0.10 |
| Main - C71.9 brain, unspecified | 0.07 | 0.09 | 0.09 |
| Main - J84.1 other interstitial pulmonary diseases with fibrosis | 0.09 | 0.10 | 0.09 |
| Sec. - G12.2 motor neuron disease | 0.10 | 0.12 | 0.09 |
| Sec. - C79.3 secondary malignant neoplasm of brain and cerebral meninges | 0.06 | 0.08 | 0.08 |
| Main - R91 abnormal findings on diagnostic imaging of lung | 0.06 | 0.08 | 0.08 |
| Sec. - R11 nausea and vomiting | 0.07 | 0.08 | 0.08 |
| Sec. - G30.9 Alzheimer's disease, unspecified | 0.05 | 0.06 | 0.08 |
| Sec. - G91.9 hydrocephalus, unspecified | 0.06 | 0.08 | 0.08 |
| Sec. - E11.9 non-insulin-dependent diabetes mellites without complications | 0.06 | 0.07 | 0.08 |
| Sec. - G93.1 anoxic brain damage, not elsewhere classified | 0.05 | 0.06 | 0.07 |
| Main - I60.9 subarachnoid hemorrhage, unspecified | 0.07 | 0.08 | 0.07 |
| Sec. - I63.9 Cerebral infarction, unspecified | 0.06 | 0.07 | 0.07 |
| Main - I21.0 acute transmural myocardial infarction of anterior wall | 0.06 | 0.07 | 0.05 |
| Main - I21.9 acute myocardial infarction, unspecified | 0.06 | 0.07 | 0.05 |
| **J - Hospital diagnoses Total** | **45.62** | **54.28** | **49.61** |
| **Grand Total** | **84.04** | **100.00** | **100.00** |

Abbreviations: A level, advanced level; AS level, advanced subsidiary level; BMI, body mass index; CSE, certificate of secondary education; NOS, not otherwise specified; Sec., secondary diagnosis.

^a^ International classification of diseases (ICD) codes are given for diagnoses. Predictor names are modified by adding additional text after a ‘—’ for some predictors to reflect how higher value(s) are coded.

^b^ SHAP values (normalized for 100%) from a GBDT model with all predictors in its input totaling 84%,

^c^ SHAP values adjusted for a total of 100%

^d^ SHAP values from a GBDT model with only important predictors in its input

**Supplementary Table S4.** Ranking of predictors based on SHAP (SHapley Additive exPlanation) values, hazard ratios (HR) and *P*-values from unadjusted and adjusted Cox models are also reported. The table is sorted on *P*-values from the adjusted Cox models.

| **Category** | **Predictor ID** | **Predictor name** ^a^ | **Ranking based on SHAP values** | **Unadjusted hazard ratio** | **Unadjusted hazard ratio 95%**  **confidence interval lower** | **Unadjusted hazard ratio 95%**  **confidence interval upper** | **Unadjusted Cox *P*-value** | **Adjusted hazard ratio** | **Adjusted hazard ratio 95%**  **confidence interval lower** | **Adjusted hazard ratio 95%**  **confidence interval upper** | **Adjusted Cox *P*-value** |
| --- | --- | --- | --- | --- | --- | --- | --- | --- | --- | --- | --- |
| J - Hospital diagnoses | x41204__Z515 | Sec. - 251.5 palliative care | 1 | 82.74 | 76.08 | 89.98 | 2.17E-2312 | 62.36 | 57.06 | 68.14 | 4.07E-1812 |
| J - Hospital diagnoses | x41204__C787 | Sec. - C78.7 secondary malignant neoplasm of liver | 8 | 65.95 | 59.87 | 72.65 | 2.14E-1566 | 49.91 | 45.19 | 55.13 | 1.60E-1292 |
| J - Hospital diagnoses | x41204__Z511 | Sec. - Z51.1 chemotherapy session for neoplasm | 5 | 18.40 | 17.01 | 19.90 | 1.73E-1155 | 17.02 | 15.70 | 18.44 | 6.28E-1039 |
| J - Hospital diagnoses | x41204__C780 | Sec. - C78.0 secondary malignant neoplasm of lung | 34 | 59.92 | 53.61 | 66.97 | 8.35E-1133 | 47.63 | 42.52 | 53.35 | 3.03E-970 |
| J - Hospital diagnoses | x41204__C795 | Sec. - C79.5 Sec. malignant neoplasm of bone and bone marrow | 9 | 55.88 | 50.05 | 62.38 | 2.65E-1117 | 39.91 | 35.64 | 44.68 | 3.38E-890 |
| J - Hospital diagnoses | x40009 | Reported occurrences of cancer | 26 |  |  |  | 4.04E-806 |  |  |  | 2.66E-677 |
| J - Hospital diagnoses | x40012__3 | Malignant, primary site cancer tumor | 4 | 10.87 | 10.09 | 11.70 | 1.65E-864 | 9.00 | 8.34 | 9.73 | 9.26E-680 |
| J - Hospital diagnoses | x40008 | Age at cancer diagnosis | 2 |  |  |  | 6.69E-863 |  |  |  | 4.00E-662 |
| J - Hospital diagnoses | x40019 | Cancer report format | 91 |  |  |  | 8.37E-770 |  |  |  | 6.14E-646 |
| J - Hospital diagnoses | x41204__C786 | Sec. - C78.6 Sec. malignant neoplasm of retroperitoneum and peritoneum | 50 | 42.02 | 36.48 | 48.41 | 1.19E-584 | 38.61 | 33.40 | 44.62 | 3.28E-534 |
| J - Hospital diagnoses | x41204__J90 | Sec. - J90 pleural effusion, not elsewhere classified | 25 | 18.26 | 16.45 | 20.27 | 3.26E-647 | 12.80 | 11.51 | 14.24 | 8.10E-481 |
| J - Hospital diagnoses | x41202__C349 | Main - C34.9 bronchus or lung, unspecified | 55 | 47.50 | 41.12 | 54.87 | 7.12E-601 | 29.93 | 25.80 | 34.72 | 3.55E-439 |
| J - Hospital diagnoses | x41204__N179 | Sec. - N17.9 acute renal failure, unspecified | 12 | 18.75 | 16.89 | 20.82 | 5.12E-658 | 11.65 | 10.46 | 12.98 | 2.02E-433 |
| J - Hospital diagnoses | x41204__C793 | Sec. - C79.3 secondary malignant neoplasm of brain and cerebral meninges | 177 | 61.13 | 51.52 | 72.55 | 3.26E-484 | 49.74 | 41.78 | 59.23 | 1.86E-420 |
| J - Hospital diagnoses | x41204__R18 | Sec. - R18 ascites | 47 | 25.44 | 21.88 | 29.57 | 7.19E-388 | 19.85 | 17.05 | 23.10 | 2.26E-325 |
| J - Hospital diagnoses | x41204__Z518 | Sec. - Z51.8 other specified medical care | 42 | 33.72 | 28.51 | 39.89 | 1.40E-368 | 26.83 | 22.64 | 31.81 | 2.64E-314 |
| J - Hospital diagnoses | x40011__8140 | Histology of cancer tumor - adenocarcinoma-NOS | 56 | 9.20 | 8.43 | 10.05 | 5.47E-538 | 5.79 | 5.27 | 6.35 | 3.89E-299 |
| J - Hospital diagnoses | x41204__J181 | Sec. - J18.1 lobar pneumonia, unspecified | 71 | 17.80 | 15.55 | 20.37 | 7.69E-382 | 12.10 | 10.55 | 13.88 | 1.65E-279 |
| J - Hospital diagnoses | x41204__A419 | Sec. - A41.9 septicemia, unspecified | 117 | 19.96 | 17.15 | 23.24 | 9.50E-326 | 15.66 | 13.43 | 18.24 | 6.41E-272 |
| J - Hospital diagnoses | x41204__J969 | Sec. - J96.9 respiratory failure, unspecified | 49 | 20.78 | 17.89 | 24.12 | 1.41E-346 | 13.79 | 11.84 | 16.06 | 3.50E-249 |
| A - Baseline characteristics | x21022 | Age at recruitment | 3 | 1.86 | 1.79 | 1.92 | 1.63E-248 | 1.86 | 1.79 | 1.92 | 1.63E-248 |
| J - Hospital diagnoses | x41204__Z513 | Sec. - Z51.3 blood transfusion without reported diagnosis | 155 | 22.33 | 18.91 | 26.38 | 1.58E-292 | 17.34 | 14.65 | 20.52 | 5.54E-242 |
| J - Hospital diagnoses | x41202__J90 | Main - J90 pleural effusion, not elsewhere classified | 138 | 21.90 | 18.69 | 25.66 | 1.73E-318 | 14.99 | 12.76 | 17.60 | 2.75E-238 |
| J - Hospital diagnoses | x41204__D649 | Sec. - D64.9 anemia, unspecified | 88 | 7.87 | 7.07 | 8.76 | 1.00E-300 | 6.26 | 5.61 | 6.99 | 6.59E-236 |
| J - Hospital diagnoses | x41202__C793 | Main - C79.3 Sec. malignant neoplasm of brain and cerebral meninges | 145 | 51.82 | 41.41 | 64.84 | 3.99E-261 | 44.09 | 35.15 | 55.31 | 5.06E-235 |
| J - Hospital diagnoses | x41204__N390 | Sec. - N39.0 urinary tract infection, site not specified | 60 | 7.92 | 7.06 | 8.90 | 3.27E-268 | 6.16 | 5.47 | 6.92 | 3.24E-202 |
| J - Hospital diagnoses | x41204__R11 | Sec. - R11 nausea and vomiting | 181 | 5.91 | 5.28 | 6.62 | 5.14E-210 | 5.56 | 4.96 | 6.23 | 3.31E-191 |
| J - Hospital diagnoses | x41204__E872 | Sec. - E87.2 acidosis | 62 | 22.30 | 18.59 | 26.76 | 7.95E-245 | 15.80 | 13.12 | 19.02 | 1.31E-186 |
| J - Hospital diagnoses | x41204__Z864 | Sec. - Z86.4 personal history of psychoactive substance abuse | 30 | 4.93 | 4.53 | 5.35 | 3.35E-309 | 3.63 | 3.32 | 3.96 | 1.53E-180 |
| J - Hospital diagnoses | x41204__J22 | Sec. - J22 unspecified acute lower respiratory infection | 63 | 9.81 | 8.60 | 11.20 | 8.62E-253 | 6.88 | 6.01 | 7.86 | 1.11E-174 |
| J - Hospital diagnoses | x41204__J189 | Sec. - J18.9 pneumonia, unspecified | 170 | 16.50 | 13.93 | 19.54 | 3.17E-231 | 11.52 | 9.70 | 13.67 | 1.50E-171 |
| J - Hospital diagnoses | x41202__J181 | Main - J18.1 lobar pneumonia, unspecified | 119 | 9.81 | 8.55 | 11.25 | 1.52E-233 | 6.92 | 6.02 | 7.96 | 7.82E-164 |
| G - Self-reported diseases | x134 | Number of self-reported cancers | 29 | 2.79 | 2.62 | 2.97 | 3.45E-222 | 2.49 | 2.33 | 2.66 | 1.04E-163 |
| J - Hospital diagnoses | x41204__Z867 | Sec. - Z86.7 personal history of diseases of the circulatory system | 169 | 5.32 | 4.84 | 5.86 | 6.93E-257 | 3.81 | 3.46 | 4.20 | 1.45E-158 |
| J - Hospital diagnoses | x40011__9440 | Histology of cancer tumor - glioblastoma-NOS | 76 | 56.88 | 43.10 | 75.08 | 3.91E-179 | 45.51 | 34.37 | 60.26 | 1.76E-156 |
| J - Hospital diagnoses | x40011__8000 | Histology of cancer tumor - neoplasm | 154 | 11.55 | 9.73 | 13.70 | 3.88E-173 | 10.93 | 9.16 | 13.06 | 8.27E-154 |
| I - Health and medical history | x2178 | Overall health rating—poorer | 16 |  |  |  | 2.33E-178 |  |  |  | 1.23E-145 |
| J - Hospital diagnoses | x41202__D649 | Main - D64.9 anemia, unspecified | 147 | 7.22 | 6.31 | 8.26 | 2.40E-181 | 5.94 | 5.18 | 6.80 | 3.12E-145 |
| B - Sociodemographics | x6146__100 | Attendance/disability/mobility allowance—none | 31 | 0.23 | 0.21 | 0.25 | 3.31E-232 | 0.30 | 0.28 | 0.33 | 2.02E-143 |
| J - Hospital diagnoses | x41204__I469 | Sec. - I46.9 cardiac arrest, unspecified | 45 | 46.35 | 36.16 | 59.40 | 9.27E-202 | 25.29 | 19.66 | 32.54 | 2.81E-139 |
| F - Psychosocial factors | x6145__1 | Illness, injury, bereavement, stress in last 2 years - serious illness, injury, or assault to yourself | 152 | 3.20 | 2.94 | 3.49 | 1.64E-157 | 2.97 | 2.73 | 3.24 | 1.61E-136 |
| H - Medications & Operations | x137 | Number of treatments/medications taken | 54 | 1.17 | 1.16 | 1.18 | 4.03E-274 | 1.13 | 1.12 | 1.14 | 1.92E-135 |
| J - Hospital diagnoses | x41204__I959 | Sec. - I95.9 hypotension, unspecified | 73 | 9.01 | 7.78 | 10.44 | 9.63E-189 | 6.54 | 5.64 | 7.59 | 8.94E-135 |
| B - Sociodemographics | x6146__2 | Attendance/disability/mobility allowance - disability living allowance | 94 | 4.36 | 3.95 | 4.80 | 4.63E-194 | 3.53 | 3.19 | 3.91 | 2.18E-132 |
| J - Hospital diagnoses | x41204__R410 | Sec. - R41.0 disorientation, unspecified | 161 | 14.47 | 12.21 | 17.16 | 1.59E-207 | 8.65 | 7.27 | 10.29 | 5.16E-131 |
| J - Hospital diagnoses | x41204__I48 | Sec. - I48 atrial fibrillation and flutter | 23 | 6.27 | 5.67 | 6.94 | 1.46E-274 | 3.69 | 3.32 | 4.10 | 1.43E-129 |
| J - Hospital diagnoses | x41204__E871 | Sec. - E87.1 hypo-osmolality and hyponatremia | 165 | 12.65 | 10.58 | 15.12 | 7.79E-171 | 8.71 | 7.27 | 10.43 | 1.35E-122 |
| J - Hospital diagnoses | x41202__J22 | Main - J22 unspecified acute lower respiratory infection | 171 | 7.45 | 6.43 | 8.62 | 2.85E-159 | 5.69 | 4.91 | 6.60 | 3.76E-118 |
| J - Hospital diagnoses | x40011__8010 | Histology of cancer tumor - carcinoma-NOS | 157 | 6.55 | 5.53 | 7.75 | 9.75E-106 | 7.31 | 6.16 | 8.67 | 1.08E-114 |
| J - Hospital diagnoses | x41204__I500 | Sec. - I50.0 congestive heart failure | 101 | 13.26 | 11.23 | 15.65 | 9.77E-205 | 7.12 | 6.01 | 8.43 | 1.45E-113 |
| J - Hospital diagnoses | x41204__R945 | Sec. - R94.5 abnormal results of liver function studies | 162 | 7.62 | 6.43 | 9.02 | 2.41E-122 | 6.66 | 5.62 | 7.90 | 6.92E-106 |
| J - Hospital diagnoses | x41202__C719 | Main - C71.9 brain, unspecified | 174 | 45.97 | 32.44 | 65.15 | 1.14E-102 | 41.62 | 29.24 | 59.25 | 3.79E-95 |
| J - Hospital diagnoses | x41204__I10 | Sec. - I10 essential (primary) hypertension | 21 | 3.24 | 3.01 | 3.48 | 5.90E-217 | 2.22 | 2.06 | 2.40 | 1.63E-91 |
| J - Hospital diagnoses | x41204__I501 | Sec. - I50.1 left ventricular failure | 120 | 9.12 | 7.81 | 10.66 | 2.86E-170 | 5.16 | 4.40 | 6.05 | 3.29E-91 |
| B - Sociodemographics | x6142__4 | Current employment status - unable to work because of sickness or disability | 116 | 3.19 | 2.85 | 3.56 | 3.32E-93 | 3.35 | 2.98 | 3.77 | 2.52E-90 |
| I - Health and medical history | x2188 | Long-standing illness, disability, or infirmity | 14 | 2.71 | 2.51 | 2.92 | 2.45E-152 | 2.17 | 2.01 | 2.34 | 3.22E-90 |
| C - Lifestyle and environment | x924 | Usual walking pace | 19 |  |  |  | 2.17E-145 |  |  |  | 7.29E-88 |
| J - Hospital diagnoses | x41204__J841 | Sec. - J84.1 other interstitial pulmonary diseases with fibrosis | 69 | 16.50 | 13.08 | 20.81 | 1.27E-123 | 10.14 | 8.02 | 12.81 | 1.04E-83 |
| J - Hospital diagnoses | x41202__C509 | Main - C50.9 breast, unspecified | 72 | 3.61 | 3.10 | 4.21 | 2.28E-60 | 4.83 | 4.10 | 5.68 | 1.35E-80 |
| J - Hospital diagnoses | x41204__E119 | Sec. - E11.9 non-insulin-dependent diabetes mellites without complications | 183 | 3.94 | 3.57 | 4.35 | 4.75E-162 | 2.62 | 2.37 | 2.90 | 4.65E-77 |
| A - Baseline characteristics | x31 | Sex—male | 6 | 1.95 | 1.81 | 2.10 | 2.76E-71 |  |  |  | 2.76E-71 |
| J - Hospital diagnoses | x41204__J690 | Sec. - J69.0 pneumonitis due to food and vomit | 149 | 21.39 | 16.03 | 28.54 | 2.50E-96 | 13.58 | 10.16 | 18.16 | 2.35E-69 |
| J - Hospital diagnoses | x41202__J841 | Main - J84.1 other interstitial pulmonary diseases with fibrosis | 175 | 22.37 | 16.24 | 30.80 | 1.02E-80 | 17.41 | 12.62 | 24.00 | 4.53E-68 |
| C - Lifestyle and environment | x20116 | Smoking status—never, previous, current | 15 |  |  |  | 6.94E-95 |  |  |  | 2.93E-67 |
| J - Hospital diagnoses | x41204__R568 | Sec. - R56.8 other and unspecified convulsions | 85 | 11.00 | 8.59 | 14.10 | 2.83E-80 | 8.99 | 7.01 | 11.53 | 4.64E-67 |
| J - Hospital diagnoses | x41202__G122 | Main - G12.2 motor neuron disease | 86 | 35.64 | 24.04 | 52.84 | 9.30E-71 | 29.07 | 19.57 | 43.19 | 1.48E-62 |
| J - Hospital diagnoses | x41204__R634 | Sec. - R63.4 abnormal weight loss | 99 | 5.11 | 4.33 | 6.01 | 8.81E-85 | 4.05 | 3.44 | 4.78 | 3.65E-62 |
| J - Hospital diagnoses | x41204__F102 | Sec. - F10.2 dependence syndrome | 67 | 7.98 | 6.46 | 9.85 | 6.80E-83 | 6.16 | 4.97 | 7.64 | 1.11E-61 |
| I - Health and medical history | x2492 | Taking other prescription medications | 92 | 2.43 | 2.25 | 2.62 | 1.40E-118 | 1.92 | 1.77 | 2.08 | 5.29E-61 |
| J - Hospital diagnoses | x41204__I460 | Sec. - I46.0 cardiac arrest with successful resuscitation | 133 | 18.20 | 13.64 | 24.28 | 1.30E-86 | 11.29 | 8.45 | 15.09 | 2.68E-60 |
| C - Lifestyle and environment | x1239 | Current tobacco smoking—more frequently | 10 |  |  |  | 1.56E-60 |  |  |  | 2.37E-59 |
| J - Hospital diagnoses | x41202__R91 | Main - R91 abnormal findings on diagnostic imaging of lung | 179 | 9.38 | 7.53 | 11.68 | 7.80E-89 | 6.16 | 4.94 | 7.68 | 1.54E-58 |
| G - Self-reported diseases | x135 | Number of self-reported non-cancer illnesses | 102 | 1.19 | 1.17 | 1.21 | 2.81E-139 | 1.14 | 1.12 | 1.15 | 3.92E-58 |
| J - Hospital diagnoses | x41204__G122 | Sec. - G12.2 motor neuron disease | 176 | 31.07 | 20.96 | 46.07 | 1.40E-65 | 23.66 | 15.91 | 35.20 | 6.02E-55 |
| J - Hospital diagnoses | x41202__R69 | Main - R69 unknown and unspecified causes of morbidity | 132 | 3.30 | 2.87 | 3.80 | 3.26E-62 | 3.05 | 2.65 | 3.52 | 3.31E-53 |
| J - Hospital diagnoses | x40011__8500 | Histology of cancer tumor - duct adenocarcinoma | 74 | 2.54 | 2.18 | 2.96 | 3.06E-33 | 3.37 | 2.87 | 3.96 | 6.89E-50 |
| I - Health and medical history | x2473 | Other serious medical condition/disability diagnosed by doctor | 118 | 2.12 | 1.96 | 2.29 | 2.76E-74 | 1.83 | 1.69 | 1.97 | 4.82E-49 |
| J - Hospital diagnoses | x41204__F329 | Sec. - F32.9 depressive episode, unspecified | 109 | 2.87 | 2.50 | 3.30 | 3.80E-50 | 2.85 | 2.48 | 3.28 | 1.43E-48 |
| J - Hospital diagnoses | x41204__F101 | Sec. - F10.1 harmful use | 103 | 5.93 | 4.89 | 7.20 | 4.56E-73 | 4.30 | 3.53 | 5.23 | 5.22E-48 |
| B - Sociodemographics | x6142__1 | In paid employment or self-employed | 7 | 0.32 | 0.30 | 0.35 | 7.72E-174 | 0.52 | 0.47 | 0.57 | 3.83E-44 |
| D - Physical measurements | x47 | Hand grip strength (right) | 61 | 0.92 | 0.89 | 0.96 | 1.00E-05 | 0.71 | 0.67 | 0.74 | 2.29E-43 |
| I - Health and medical history | x2316 | Wheeze or whistling in the chest in last year | 121 | 1.98 | 1.83 | 2.14 | 4.27E-62 | 1.76 | 1.62 | 1.90 | 1.26E-42 |
| J - Hospital diagnoses | x41204__G309 | Sec. - G30.9 Alzheimer's disease, unspecified | 180 | 15.97 | 11.39 | 22.39 | 4.86E-58 | 7.73 | 5.49 | 10.87 | 6.83E-32 |
| I - Health and medical history | x6152__6 | Current employment status - doing unpaid or voluntary work | 107 | 3.88 | 3.33 | 4.53 | 9.12E-67 | 2.54 | 2.17 | 2.97 | 1.16E-31 |
| I - Health and medical history | x6150__1 | Vascular/heart problems diagnosed by doctor - heart attack | 44 | 3.91 | 3.43 | 4.45 | 1.30E-92 | 2.18 | 1.91 | 2.50 | 3.55E-30 |
| A - Baseline characteristics | x189 | Townsend deprivation index | 77 | 1.23 | 1.19 | 1.28 | 4.96E-30 | 1.23 | 1.19 | 1.28 | 4.96E-30 |
| I - Health and medical history | x2443 | Diabetes diagnosed by doctor | 43 | 2.84 | 2.55 | 3.16 | 1.08E-63 | 1.97 | 1.76 | 2.19 | 2.04E-29 |
| C - Lifestyle and environment | x6164__2 | Physical activity in last 4 weeks - other exercises (egg: swimming, cycling, keep fit, bowling) | 32 | 0.55 | 0.51 | 0.59 | 1.74E-52 | 0.65 | 0.60 | 0.70 | 4.02E-27 |
| J - Hospital diagnoses | x41202__I639 | Main - I63.9 cerebral infarction, unspecified | 168 | 5.44 | 4.34 | 6.81 | 2.98E-49 | 3.45 | 2.75 | 4.33 | 6.87E-27 |
| J - Hospital diagnoses | x41204__G931 | Sec. - G93.1 anoxic brain damage, not elsewhere classified | 186 | 32.03 | 18.95 | 54.16 | 2.72E-38 | 18.04 | 10.59 | 30.70 | 1.67E-26 |
| D - Physical measurements | x3088 | Contra-indications for spirometry | 153 |  |  |  | 1.10E-45 |  |  |  | 2.80E-26 |
| H - Medications & Operations | x136 | Number of operations, self-reported | 190 | 1.14 | 1.12 | 1.16 | 1.79E-35 | 1.13 | 1.10 | 1.15 | 9.16E-26 |
| B - Sociodemographics | x728 | No. of vehicles in household | 18 | 0.67 | 0.64 | 0.70 | 5.08E-67 | 0.76 | 0.72 | 0.80 | 1.01E-25 |
| J - Hospital diagnoses | x41204__F03 | Sec. - F03 unspecified dementia | 144 | 12.27 | 8.71 | 17.30 | 1.59E-46 | 6.16 | 4.36 | 8.71 | 7.57E-25 |
| D - Physical measurements | x48 | Waist circumference | 13 | 1.45 | 1.40 | 1.50 | 2.26E-89 | 1.23 | 1.18 | 1.28 | 8.72E-22 |
| I - Health and medical history | x6150__100 | No vascular/heart problems diagnosed by doctor | 90 | 0.48 | 0.45 | 0.52 | 1.21E-86 | 0.70 | 0.65 | 0.75 | 9.17E-21 |
| J - Hospital diagnoses | x41204__I639 | Sec. - I63.9 Cerebral infarction, unspecified | 188 | 12.11 | 8.10 | 18.09 | 4.67E-34 | 6.70 | 4.47 | 10.03 | 2.62E-20 |
| J - Hospital diagnoses | x41204__G919 | Sec. - G91.9 hydrocephalus, unspecified | 182 | 9.71 | 6.11 | 15.43 | 7.07E-22 | 8.76 | 5.51 | 13.95 | 5.58E-20 |
| C - Lifestyle and environment | x20160 | Ever smoked | 66 | 1.67 | 1.55 | 1.81 | 1.49E-38 | 1.43 | 1.32 | 1.55 | 1.86E-18 |
| I - Health and medical history | x6149__6 | Mouth/teeth dental problems - dentures | 53 | 2.26 | 2.09 | 2.44 | 9.05E-92 | 1.44 | 1.33 | 1.57 | 8.54E-18 |
| J - Hospital diagnoses | x41202__I619 | Main - I61.9 intracerebral hemorrhage, unspecified | 166 | 9.89 | 6.50 | 15.04 | 9.47E-27 | 6.21 | 4.08 | 9.46 | 1.69E-17 |
| C - Lifestyle and environment | x943 | Frequency of stair climbing in last 4 weeks | 40 |  |  |  | 4.92E-38 |  |  |  | 2.83E-17 |
| E - Cognitive function | x20023 | Mean time to correctly identify matches | 22 | 1.37 | 1.32 | 1.42 | 9.17E-64 | 1.18 | 1.13 | 1.23 | 1.28E-16 |
| B - Sociodemographics | x709 | Number in household | 17 | 0.70 | 0.67 | 0.73 | 3.82E-76 | 0.85 | 0.81 | 0.88 | 7.43E-16 |
| C - Lifestyle and environment | x2267 | Use of sun/UV protection—always | 33 |  |  |  | 3.59E-41 |  |  |  | 6.22E-15 |
| I - Health and medical history | x1835 | Mother still alive | 46 | 0.38 | 0.35 | 0.42 | 3.86E-114 | 0.68 | 0.62 | 0.75 | 2.05E-14 |
| C - Lifestyle and environment | x1448 | Bread type | 98 |  |  |  | 5.86E-27 |  |  |  | 6.65E-14 |
| J - Hospital diagnoses | x41204__G20 | Sec. - G20 Parkinson's disease | 125 | 5.42 | 3.95 | 7.43 | 1.07E-25 | 3.32 | 2.42 | 4.56 | 1.17E-13 |
| I - Health and medical history | x6154__100 | Medication for pain relief, constipation, heartburn—none | 83 | 0.67 | 0.62 | 0.72 | 3.28E-27 | 0.75 | 0.70 | 0.81 | 1.34E-13 |
| F - Psychosocial factors | x1960 | Fed-up feelings | 115 | 1.16 | 1.08 | 1.25 | 1.04E-04 | 1.32 | 1.22 | 1.42 | 9.28E-13 |
| C - Lifestyle and environment | x6164__5 | Types of physical activity in last 4 weeks - Heavy DIY (egg: weeding, lawn mowing, carpentry, digging) | 105 | 0.83 | 0.77 | 0.90 | 2.56E-06 | 0.75 | 0.69 | 0.81 | 1.53E-12 |
| D - Physical measurements | x20015 | Sitting height | 39 | 0.98 | 0.95 | 1.02 | 2.97E-01 | 0.85 | 0.81 | 0.89 | 6.14E-11 |
| J - Hospital diagnoses | x40006__C61 | C61 malignant neoplasm of prostate | 38 | 3.46 | 2.95 | 4.05 | 1.50E-52 | 1.71 | 1.45 | 2.01 | 1.73E-10 |
| J - Hospital diagnoses | x41202__I609 | Main - I60.9 subarachnoid hemorrhage, unspecified | 187 | 5.45 | 3.28 | 9.05 | 5.73E-11 | 4.91 | 2.95 | 8.16 | 8.32E-10 |
| I - Health and medical history | x1797 | Father still alive | 108 | 0.33 | 0.29 | 0.38 | 9.53E-86 | 0.67 | 0.58 | 0.76 | 1.59E-09 |
| C - Lifestyle and environment | x1478 | Salt added to food | 141 |  |  |  | 5.87E-15 |  |  |  | 6.51E-09 |
| I - Health and medical history | x2306 | Weight change compared with 1 year ago—lost, same, gained | 151 |  |  |  | 2.05E-05 |  |  |  | 2.80E-08 |
| C - Lifestyle and environment | x1349 | Processed meat intake | 59 |  |  |  | 5.13E-23 |  |  |  | 5.19E-08 |
| C - Lifestyle and environment | x1418 | Milk type used | 167 |  |  |  | 2.63E-13 |  |  |  | 6.65E-08 |
| B - Sociodemographics | x670 | Type of accommodation lived in—house, flat, mobile, sheltered, care home | 148 |  |  |  | 1.13E-20 |  |  |  | 9.90E-08 |
| E - Cognitive function | x400 | Time to complete round | 35 | 1.33 | 1.28 | 1.38 | 7.58E-51 | 1.11 | 1.07 | 1.16 | 1.01E-07 |
| I - Health and medical history | x6149__100 | Mouth/teeth dental problems—none | 184 | 0.69 | 0.64 | 0.75 | 6.05E-23 | 0.82 | 0.76 | 0.88 | 1.13E-07 |
| I - Health and medical history | x6154__3 | Medication for pain relief, constipation, heartburn - paracetamol | 52 | 1.14 | 1.05 | 1.24 | 2.25E-03 | 1.26 | 1.16 | 1.37 | 1.43E-07 |
| C - Lifestyle and environment | x6164__4 | Light DIY physical activity in last 4 weeks | 84 | 0.76 | 0.71 | 0.82 | 2.28E-13 | 0.82 | 0.76 | 0.88 | 1.61E-07 |
| C - Lifestyle and environment | x1488 | Tea intake | 51 |  |  |  | 1.07E-07 |  |  |  | 2.41E-07 |
| C - Lifestyle and environment | x6144__5 | Eat eggs, diary, wheat, sugar | 95 | 0.67 | 0.62 | 0.73 | 3.03E-23 | 0.81 | 0.75 | 0.88 | 3.46E-07 |
| J - Hospital diagnoses | x41202__I219 | Main - I21.9 acute myocardial infarction, unspecified | 192 | 2.90 | 2.28 | 3.68 | 2.64E-18 | 1.85 | 1.45 | 2.35 | 5.48E-07 |
| C - Lifestyle and environment | x6164__3 | Types of physical activity in last 4 weeks - strenuous sports | 140 | 0.50 | 0.43 | 0.59 | 2.44E-16 | 0.65 | 0.55 | 0.77 | 5.62E-07 |
| I - Health and medical history | x6179__2 | Mineral and other dietary supplements - glucosamine | 82 | 0.84 | 0.76 | 0.92 | 4.12E-04 | 0.78 | 0.71 | 0.87 | 1.46E-06 |
| B - Sociodemographics | x6138__2 | Qualifications - A levels/AS levels or equivalent | 146 | 0.59 | 0.54 | 0.65 | 3.48E-26 | 0.79 | 0.71 | 0.87 | 1.54E-06 |
| C - Lifestyle and environment | x864 | Number of days/week walked 10+ minutes | 126 | 0.98 | 0.97 | 1.00 | 9.14E-02 | 0.96 | 0.94 | 0.97 | 2.93E-06 |
| D - Physical measurements | x23120 | Arm fat mass (right) | 106 | 1.04 | 1.00 | 1.08 | 3.83E-02 |  |  |  | 3.24E-06 |
| J - Hospital diagnoses | x41202__I210 | Main - I21.0 acute transmural myocardial infarction of anterior wall | 191 | 3.53 | 2.53 | 4.93 | 1.17E-13 | 2.19 | 1.57 | 3.07 | 4.15E-06 |
| J - Hospital diagnoses | x40012__2 | Behavior of cancer tumor - carcinoma in situ cancer tumor | 57 | 1.41 | 1.17 | 1.72 | 4.35E-04 | 1.56 | 1.28 | 1.89 | 7.49E-06 |
| J - Hospital diagnoses | x41202__Z080 | Main - Z08.0 follow-up exam. after surgery for malignant neoplasm | 65 | 2.70 | 2.14 | 3.41 | 8.77E-17 | 1.70 | 1.34 | 2.15 | 9.83E-06 |
| C - Lifestyle and environment | x1289 | Cooked vegetable intake | 89 |  |  |  | 8.83E-08 |  |  |  | 1.38E-05 |
| I - Health and medical history | x6179__1 | Mineral and other dietary supplements - fish oil (including cod liver oil) | 189 | 0.98 | 0.91 | 1.06 | 6.89E-01 | 0.84 | 0.78 | 0.91 | 1.68E-05 |
| B - Sociodemographics | x21000 | Ethnic background | 28 |  |  |  | 1.87E-08 |  |  |  | 1.69E-05 |
| I - Health and medical history | x6159__100 | Pain type(s) experienced in last month—none | 130 | 0.85 | 0.79 | 0.91 | 1.99E-05 | 0.86 | 0.79 | 0.92 | 7.29E-05 |
| D - Physical measurements | x21001 | Body mass index (BMI) | 41 | 1.16 | 1.12 | 1.21 | 4.64E-16 | 1.08 | 1.03 | 1.12 | 2.30E-04 |
| I - Health and medical history | x6152__9 | Hay fever, allergic rhinitis or eczema diagnosed by doctor | 142 | 0.68 | 0.62 | 0.75 | 4.38E-15 | 0.83 | 0.76 | 0.92 | 2.35E-04 |
| B - Sociodemographics | x6142__5 | Current employment status - unemployed | 193 | 1.31 | 1.03 | 1.66 | 2.54E-02 | 1.54 | 1.21 | 1.96 | 4.41E-04 |
| C - Lifestyle and environment | x1369 | Beef intake | 78 |  |  |  | 6.33E-10 |  |  |  | 6.83E-04 |
| I - Health and medical history | x2463 | Fractured/broken bones in last 5 years | 112 | 1.18 | 1.05 | 1.32 | 6.64E-03 | 1.23 | 1.09 | 1.38 | 7.29E-04 |
| C - Lifestyle and environment | x1717 | Skin color—less fair | 135 |  |  |  | 3.70E-06 |  |  |  | 7.79E-04 |
| B - Sociodemographics | x6139__3 | Gas or solid-fuel cooking/heating - an open solid fuel fire that you use regularly in winter time | 114 | 0.68 | 0.58 | 0.79 | 7.87E-07 | 0.78 | 0.67 | 0.91 | 1.74E-03 |
| C - Lifestyle and environment | x1389 | Pork intake | 113 |  |  |  | 3.39E-08 |  |  |  | 1.78E-03 |
| I - Health and medical history | x6179__100 | Mineral and other dietary supplements—none | 185 | 1.00 | 0.93 | 1.07 | 9.30E-01 | 1.13 | 1.05 | 1.21 | 1.82E-03 |
| D - Physical measurements | x23111 | Leg fat percentage (right) | 11 | 0.87 | 0.84 | 0.90 | 1.64E-13 | 1.10 | 1.04 | 1.17 | 1.95E-03 |
| B - Sociodemographics | x6138__4 | Qualifications - CSEs or equivalent | 136 | 0.45 | 0.39 | 0.52 | 7.57E-25 | 0.78 | 0.67 | 0.92 | 2.49E-03 |
| I - Health and medical history | x1883 | Number of full sisters | 96 | 1.06 | 1.03 | 1.09 | 2.28E-05 | 1.04 | 1.02 | 1.07 | 2.54E-03 |
| D - Physical measurements | x50 | Standing height | 122 | 1.10 | 1.06 | 1.14 | 5.64E-07 | 0.93 | 0.88 | 0.98 | 4.35E-03 |
| J - Hospital diagnoses | x41202__M179 | Main - M17.9 gonarthrosis, unspecified | 70 | 0.99 | 0.78 | 1.25 | 9.33E-01 | 0.71 | 0.56 | 0.90 | 5.20E-03 |
| C - Lifestyle and environment | x1518 | Hot drink temperature—less hot | 164 |  |  |  | 9.41E-04 |  |  |  | 8.35E-03 |
| C - Lifestyle and environment | x1359 | Poultry intake | 48 |  |  |  | 3.06E-12 |  |  |  | 9.20E-03 |
| I - Health and medical history | x2257 | Hearing difficulty/problems with background noise | 58 | 1.39 | 1.29 | 1.50 | 2.94E-18 | 1.10 | 1.02 | 1.19 | 9.56E-03 |
| D - Physical measurements | x49 | Hip circumference | 124 | 1.08 | 1.04 | 1.12 | 6.53E-05 | 1.05 | 1.01 | 1.09 | 1.02E-02 |
| D - Physical measurements | x21002 | Weight | 137 | 1.20 | 1.16 | 1.24 | 9.56E-23 | 1.06 | 1.01 | 1.10 | 1.04E-02 |
| D - Physical measurements | x23110 | Impedance of arm (left) | 97 | 0.81 | 0.78 | 0.84 | 6.08E-28 | 1.07 | 1.02 | 1.13 | 1.15E-02 |
| J - Hospital diagnoses | x41202__M169 | Main - M16.9 coxarthrosis, unspecified | 139 | 0.99 | 0.74 | 1.31 | 9.25E-01 | 0.71 | 0.53 | 0.94 | 1.61E-02 |
| F - Psychosocial factors | x2000 | Worry too long after embarrassment | 37 | 0.79 | 0.73 | 0.85 | 8.60E-10 | 0.91 | 0.85 | 0.98 | 1.76E-02 |
| C - Lifestyle and environment | x1647 | Country of birth (UK/elsewhere) | 150 |  |  |  | 1.74E-06 |  |  |  | 1.87E-02 |
| B - Sociodemographics | x6139__100 | Gas or solid-fuel cooking/heating—none | 159 | 1.28 | 1.17 | 1.40 | 7.30E-08 | 1.11 | 1.02 | 1.22 | 2.15E-02 |
| D - Physical measurements | x23107 | Impedance of leg (right) | 36 | 0.81 | 0.78 | 0.84 | 7.55E-28 | 0.95 | 0.92 | 0.99 | 2.32E-02 |
| I - Health and medical history | x2345 | Ever had bowel cancer screening | 20 | 1.64 | 1.52 | 1.76 | 8.11E-37 | 1.10 | 1.01 | 1.19 | 2.39E-02 |
| I - Health and medical history | x6159__3 | Pain type(s) experienced in last month - neck or shoulder pain | 131 | 1.11 | 1.02 | 1.20 | 1.81E-02 | 1.10 | 1.01 | 1.19 | 2.88E-02 |
| C - Lifestyle and environment | x1697 | Comparative height size at age 10 | 100 |  |  |  | 1.58E-01 |  |  |  | 6.66E-02 |
| F - Psychosocial factors | x2040 | Risk taking | 79 | 1.11 | 1.03 | 1.21 | 1.03E-02 | 1.08 | 0.99 | 1.18 | 6.84E-02 |
| F - Psychosocial factors | x6145__2 | Illness, injury, bereavement, stress in last 2 years - Serious illness, injury, or assault of a close relative | 64 | 0.72 | 0.63 | 0.82 | 1.23E-06 | 0.89 | 0.78 | 1.01 | 7.48E-02 |
| J - Hospital diagnoses | x41202__K409 | Main - K40.9 unilateral or unspecified inguinal hernia | 163 | 1.43 | 1.20 | 1.70 | 6.70E-05 | 0.85 | 0.71 | 1.02 | 8.12E-02 |
| B - Sociodemographics | x699 | Length of time at current address | 27 | 1.26 | 1.21 | 1.30 | 5.80E-34 | 1.03 | 0.99 | 1.07 | 9.90E-02 |
| J - Hospital diagnoses | x41204__M179 | Sec. - M17.9 gonarthrosis, unspecified | 128 | 1.43 | 1.14 | 1.79 | 1.83E-03 | 1.21 | 0.96 | 1.52 | 9.97E-02 |
| F - Psychosocial factors | x6145__3 | Illness, injury, bereavement, stress in last 2 years - Death of a close relative | 178 | 0.88 | 0.80 | 0.97 | 7.48E-03 | 0.93 | 0.85 | 1.02 | 1.22E-01 |
| I - Health and medical history | x2247 | Hearing difficulty/problems | 160 |  |  |  | 5.00E-18 |  |  |  | 1.36E-01 |
| A - Baseline characteristics | x52 | Month of birth | 87 |  |  |  | 1.81E-01 |  |  |  | 1.81E-01 |
| I - Health and medical history | x6154__2 | Medication for pain relief, constipation, heartburn - ibuprofen (e.g. Nurofen) | 158 | 0.71 | 0.63 | 0.80 | 1.26E-08 | 0.93 | 0.82 | 1.04 | 2.05E-01 |
| I - Health and medical history | x6159__1 | Pain type(s) experienced in last month - headache | 75 | 0.72 | 0.65 | 0.79 | 4.35E-11 | 0.94 | 0.85 | 1.04 | 2.08E-01 |
| J - Hospital diagnoses | x41202__K219 | Main - K21.9 gastro-esophageal reflux disease without esophagitis | 111 | 1.27 | 0.96 | 1.67 | 9.17E-02 | 1.19 | 0.91 | 1.58 | 2.10E-01 |
| B - Sociodemographics | x6139__2 | Gas or solid-fuel cooking/heating - a gas fire that you use regularly in winter time | 110 | 0.99 | 0.92 | 1.07 | 7.81E-01 | 0.95 | 0.88 | 1.03 | 2.14E-01 |
| I - Health and medical history | x1873 | Number of full brothers | 68 | 1.04 | 1.01 | 1.07 | 4.14E-03 | 1.02 | 0.99 | 1.04 | 2.25E-01 |
| I - Health and medical history | x6159__7 | Pain type(s) experienced in last month - knee pain | 81 | 1.19 | 1.10 | 1.30 | 4.24E-05 | 1.05 | 0.97 | 1.15 | 2.33E-01 |
| F - Psychosocial factors | x1940 | Irritability | 134 | 1.00 | 0.92 | 1.09 | 9.58E-01 | 1.05 | 0.97 | 1.14 | 2.55E-01 |
| J - Hospital diagnoses | x41202__C443 | Main - C44.3 Other/unspecified malignant neoplasm of skin of other and unspecified parts of face | 172 | 1.21 | 0.90 | 1.63 | 2.10E-01 | 0.84 | 0.63 | 1.14 | 2.61E-01 |
| F - Psychosocial factors | x2030 | Guilty feelings | 129 | 0.84 | 0.77 | 0.91 | 4.06E-05 | 0.95 | 0.88 | 1.04 | 2.71E-01 |
| F - Psychosocial factors | x1950 | Sensitivity / hurt feelings | 123 | 0.90 | 0.84 | 0.97 | 7.37E-03 | 1.04 | 0.97 | 1.12 | 2.91E-01 |
| J - Hospital diagnoses | x41202__K635 | Main - K63.5 polyp of colon | 104 | 1.50 | 1.21 | 1.86 | 1.92E-04 | 1.10 | 0.88 | 1.36 | 4.03E-01 |
| C - Lifestyle and environment | x1408 | Cheese intake | 143 |  |  |  | 5.68E-02 |  |  |  | 5.38E-01 |
| C - Lifestyle and environment | x1438 | Bread intake | 93 | 1.13 | 1.09 | 1.17 | 1.27E-10 | 0.99 | 0.95 | 1.03 | 6.64E-01 |
| D - Physical measurements | x23101 | Whole body fat-free mass | 80 | 1.20 | 1.16 | 1.24 | 5.55E-22 | 1.01 | 0.95 | 1.07 | 7.26E-01 |
| C - Lifestyle and environment | x1707 | Handedness—right-handed, left-handed, use both hands | 156 |  |  |  | 5.12E-01 |  |  |  | 7.97E-01 |
| F - Psychosocial factors | x1980 | Worrier / anxious feelings | 173 | 0.88 | 0.82 | 0.95 | 1.22E-03 | 1.01 | 0.93 | 1.08 | 8.92E-01 |
| E - Cognitive function | x399 | Number of incorrect matches in round | 127 | 1.10 | 1.06 | 1.14 | 2.27E-07 | 1.00 | 0.97 | 1.04 | 9.08E-01 |
| J - Hospital diagnoses | x40011__8090 | Histology of cancer tumor - Basal cell carcinoma, NOS | 24 | 1.41 | 1.18 | 1.68 | 1.38E-04 | 1.00 | 0.84 | 1.20 | 9.58E-01 |

Abbreviations: A level, advanced level; AS level, advanced subsidiary level; CSE, certificate of secondary education; NOS, not otherwise specified; Sec., secondary diagnosis.

^a^ International classification of diseases (ICD) codes are given for diagnoses. Predictor names are modified by adding additional text after a ‘—’ for some predictors to reflect how higher value(s) are coded.

^b^ adjusted for age, sex, Townsend deprivation index, assessment center and month of birth.

**Supplementary Table S5.** Features selected by logistic regression LASSO models for five different seeds for splitting the data and with a value of 0.03 for the hyper-parameter *C*. The features are ranked by the average absolute coefficients.

| **Field ID** | **Field Description ^a^** | **Absolute coefficient** | | | | | |
| --- | --- | --- | --- | --- | --- | --- | --- |
|  |  | **Seed 123** | **Seed 124** | **Seed 125** | **Seed 126** | **Seed 127** | **Average** |
| x34 | Year of birth | 3.34 | 3.19 | 2.98 | 2.96 | 3.34 | 3.16 |
| x41204__Z515 | Sec. - Z51.5 palliative care | 3.16 | 3.16 | 3.09 | 3.13 | 3.23 | 3.16 |
| x40011__9440 | Histology of cancer tumor — glioblastoma-NOS | 3.01 | 3.17 | 2.97 | 2.64 | 3.09 | 2.98 |
| x41204__I469 | Sec. - I46.9 cardiac arrest, unspecified | 2.98 | 2.93 | 3.00 | 2.74 | 2.99 | 2.93 |
| x41202__G122 | Main - G12.2 motor neuron disease | 2.72 | 2.86 | 1.84 | 2.55 | 2.01 | 2.39 |
| x41204__C787 | Sec. - C78.7 secondary malignant neoplasm of liver | 2.18 | 2.19 | 2.06 | 2.02 | 2.04 | 2.10 |
| x41204__C795 | Sec. - C79.5 Sec. malig. neoplasm of bone and bone marrow | 1.62 | 1.78 | 1.81 | 1.82 | 1.58 | 1.72 |
| x41204__Z518 | Sec. - Z51.8 other specified medical care | 1.46 | 1.56 | 1.66 | 1.70 | 1.66 | 1.61 |
| x41204__C793 | Sec. - C79.3 secondary malignant neoplasm of brain and cerebral meninges | 1.46 | 1.53 | 1.53 | 1.55 | 1.25 | 1.46 |
| x41204__C786 | Sec. - C78.6 Sec. malig. neoplasm of retroperitoneum and peritoneum | 1.36 | 1.38 | 1.28 | 1.45 | 1.39 | 1.37 |
| x40012__3 | Tumor behavior - malignant, primary site | 1.33 | 1.32 | 1.32 | 1.33 | 1.30 | 1.32 |
| x41204__C780 | Sec. - C78.0 secondary malignant neoplasm of lung | 1.20 | 1.21 | 1.36 | 1.06 | 1.33 | 1.23 |
| x41204__R18 | Sec. - R18 ascites | 1.24 | 1.30 | 1.13 | 1.05 | 1.11 | 1.16 |
| x41204__J841 | Sec. - J84.1 other interstitial pulmonary diseases with fibrosis | 1.15 | 1.21 | 1.18 | 1.04 | 1.21 | 1.16 |
| x41202__C349 | Main - C34.9 bronchus or lung, unspecified | 1.11 | 1.08 | 0.90 | 0.98 | 0.97 | 1.01 |
| x41204__J969 | Sec. - J96.9 respiratory failure, unspecified | 0.77 | 0.99 | 0.85 | 0.87 | 1.01 | 0.90 |
| x40006__C61 | C61 - malignant neoplasm of prostate | 0.74 | 0.96 | 1.04 | 0.88 | 0.85 | 0.90 |
| x41202__C259 | Main - C25.9 malignant neoplasm of pancreas, unspecified | 0.99 | 0.93 | 0.84 | 0.69 | 1.01 | 0.89 |
| x41204__N179 | Sec. - N17.9 acute renal failure, unspecified | 0.85 | 0.91 | 0.80 | 0.87 | 0.83 | 0.85 |
| x41204__E872 | Sec. - E87.2 acidosis | 0.91 | 0.85 | 0.83 | 0.82 | 0.83 | 0.85 |
| x41204__Z511 | Sec. - Z51.1 chemotherapy session for neoplasm | 0.90 | 0.80 | 0.84 | 0.83 | 0.82 | 0.84 |
| x41204__I460 | Sec. - I46.0 cardiac arrest with successful resuscitation | 0.68 | 0.69 | 0.88 | 0.68 | 1.02 | 0.79 |
| x40011__8090 | Histology of cancer tumor — Basal cell carcinoma, NOS | 0.68 | 0.72 | 0.79 | 0.65 | 0.73 | 0.71 |
| x41202__C793 | Main - C79.3 sec. malig. neoplasm of brain and cerebral meninges | 0.84 | 0.87 | 0.59 | 0.38 | 0.87 | 0.71 |
| x40006__C450 | C45.0 - mesothelioma of pleura | 0.03 | 0.97 | 0.78 | 0.82 | 0.88 | 0.70 |
| x40006__C349 | C34.9 - malignant neoplasm of unspecified part of bronchus or lung | 0.55 | 0.70 | 0.78 | 0.56 | 0.61 | 0.64 |
| x41204__F03 | Sec. - F03 unspecified dementia | 0.46 | 0.79 | 0.73 | 0.70 | 0.47 | 0.63 |
| x23115 | Leg fat percentage (left) | 0.68 | 0.44 | 0.88 | 0.51 | 0.66 | 0.63 |
| x40011__8000 | Histology of cancer tumor — neoplasm | 0.70 | 0.56 | 0.58 | 0.50 | 0.57 | 0.58 |
| x41202__C719 | Main - C71.9 brain, unspecified | 0.51 | 0.11 | 0.73 | 1.00 | 0.54 | 0.58 |
| x41204__R568 | Sec. - R56.8 other and unspecified convulsions | 0.56 | 0.61 | 0.55 | 0.66 | 0.38 | 0.55 |
| x41204__J690 | Sec. - J69.0 pneumonitis due to food and vomit | 0.72 | 0.18 | 0.39 | 0.68 | 0.57 | 0.51 |
| x41204__Z513 | Sec. - Z51.3 blood transfusion without reported diagnosis | 0.52 | 0.54 | 0.45 | 0.35 | 0.67 | 0.51 |
| x41204__C80 | Sec. - C80 malignant neoplasm, without specification of site | 0.37 | 0.42 | 0.51 | 0.46 | 0.73 | 0.50 |
| x41202__J90 | Main - J90 pleural effusion, not elsewhere classified | 0.52 | 0.47 | 0.41 | 0.47 | 0.56 | 0.49 |
| x41202__Z080 | Main - Z08.0 follow-up exam. after surgery for malig. neoplasm | 0.55 | 0.53 | 0.48 | 0.45 | 0.38 | 0.48 |
| x41204__I500 | Sec. - I50.0 congestive heart failure | 0.51 | 0.40 | 0.45 | 0.48 | 0.47 | 0.46 |
| x31 | Sex | 0.42 | 0.47 | 0.43 | 0.46 | 0.54 | 0.46 |
| x41204__J90 | Sec. - J90 pleural effusion, not elsewhere classified | 0.41 | 0.43 | 0.50 | 0.47 | 0.46 | 0.46 |
| x41204__F102 | Sec. - F10.2 dependence syndrome | 0.42 | 0.43 | 0.52 | 0.48 | 0.41 | 0.45 |
| x41204__G20 | Sec. - G20 Parkinson’s disease | 0.69 | 0.19 | 0.49 | 0.43 | 0.37 | 0.43 |
| x40011__8010 | Histology of cancer tumor — carcinoma-NOS | 0.44 | 0.42 | 0.33 | 0.44 | 0.53 | 0.43 |
| x40011__8140 | Histology of cancer tumor — adenocarcinoma-NOS | 0.28 | 0.53 | 0.49 | 0.44 | 0.42 | 0.43 |
| x41204__Z850 | Sec. - Z85.0 personal history of malignant neoplasm of digestive organs | 0.32 | 0.49 | 0.44 | 0.43 | 0.47 | 0.43 |
| x41204__I501 | Sec. - I50.1 left ventricular failure | 0.30 | 0.46 | 0.41 | 0.35 | 0.44 | 0.39 |
| x2453 | Cancer diagnosed by doctor | 0.43 | 0.38 | 0.35 | 0.37 | 0.38 | 0.38 |
| x41204__J189 | Sec. - J18.9 pneumonia, unspecified | 0.43 | 0.35 | 0.38 | 0.28 | 0.42 | 0.37 |
| x41204__A419 | Sec. - A41.9 septicemia, unspecified | 0.38 | 0.49 | 0.44 | 0.33 | 0.19 | 0.37 |
| x1239 | Current tobacco smoking | 0.38 | 0.36 | 0.37 | 0.37 | 0.37 | 0.37 |
| x41204__I48 | Sec. - I48 atrial fibrillation and flutter | 0.32 | 0.36 | 0.40 | 0.41 | 0.33 | 0.37 |
| x41202__J841 | Main - J84.1 other interstitial pulmonary diseases with fibrosis | 0.23 | 0.46 | 0.27 | 0.47 | 0.36 | 0.36 |
| x41204__J181 | Sec. - J18.1 lobar pneumonia, unspecified | 0.35 | 0.37 | 0.40 | 0.34 | 0.33 | 0.36 |
| x41202__I619 | Main - I61.9 intracerebral hemorrhage, unspecified | 0.16 | 0.38 | 0.52 | 0.28 | 0.44 | 0.36 |
| x41204__R945 | Sec. - R94.5 abnormal results of liver function studies | 0.27 | 0.32 | 0.36 | 0.32 | 0.35 | 0.32 |
| x924 | Usual walking pace | 0.29 | 0.32 | 0.37 | 0.24 | 0.32 | 0.31 |
| x41202__C509 | Main - C50.9 breast, unspecified | 0.43 | 0.34 | 0.17 | 0.33 | 0.26 | 0.30 |
| x709 | Number in household | 0.30 | 0.25 | 0.32 | 0.30 | 0.32 | 0.30 |
| x41204__R634 | Sec. - R63.4 abnormal weight loss | 0.29 | 0.28 | 0.27 | 0.33 | 0.32 | 0.30 |
| x6150__1 | Vascular/heart problems diagnosed by doctor — heart attack | 0.32 | 0.32 | 0.29 | 0.34 | 0.23 | 0.30 |
| x41204__R410 | Sec. - R41.0 disorientation, unspecified | 0.34 | 0.24 | 0.27 | 0.35 | 0.28 | 0.29 |
| x41204__F101 | Sec. - F10.1 harmful use | 0.35 | 0.31 | 0.18 | 0.24 | 0.34 | 0.29 |
| x41204__I269 | Sec. - I26.9 pulmonary embolism without acute cor pulmonale | 0.20 | 0.23 | 0.37 | 0.19 | 0.41 | 0.28 |
| x40006__C341 | C34.1 - malignant neoplasm of upper lobe, bronchus, or lung | 0.27 | 0.40 | 0.24 | 0.13 | 0.36 | 0.28 |
| x6146__100 | Getting no attendance/disability/mobility allowance | 0.27 | 0.28 | 0.23 | 0.30 | 0.28 | 0.27 |
| x41202__J690 | Main - J69.0 pneumonitis due to inhalation of food and vomit | 0.60 | 0.41 | 0.09 | 0.14 | 0.08 | 0.26 |
| x41204__C349 | Sec. - C34.9 malignant neoplasm of unspecified part of bronchus or lung. | 0.06 | 0.28 | 0.32 | 0.38 | 0.28 | 0.26 |
| x41204__G122 | Sec. - G12.2 motor neuron disease | - | - | 0.61 | - | 0.64 | 0.25 |
| x41204__I959 | Sec. - I95.9 hypotension, unspecified | 0.23 | 0.24 | 0.32 | 0.21 | 0.24 | 0.25 |
| x20116 | Smoking status | 0.23 | 0.26 | 0.25 | 0.21 | 0.27 | 0.24 |
| x41202__J189 | Main - J18.9 pneumonia, unspecified | 0.28 | 0.13 | 0.17 | 0.28 | 0.33 | 0.24 |
| x41204__D649 | Sec. - D64.9 anemia, unspecified | 0.23 | 0.19 | 0.22 | 0.28 | 0.23 | 0.23 |
| x41204__N390 | Sec. - N39.0 urinary tract infection, site not specified | 0.25 | 0.15 | 0.23 | 0.26 | 0.26 | 0.23 |
| x41204__R32 | Sec. - R32 unspecified urinary incontinence | 0.08 | 0.38 | 0.42 | 0.03 | 0.23 | 0.23 |
| x398 | Number of correct matches in round | 0.14 | 0.33 | 0.20 | 0.28 | 0.17 | 0.22 |
| x41202__M179 | Main - M17.9 gonarthrosis, unspecified | 0.26 | 0.19 | 0.18 | 0.25 | 0.21 | 0.22 |
| x6142__1 | Neither in paid employment nor self-employed | 0.23 | 0.18 | 0.22 | 0.25 | 0.20 | 0.22 |
| x41202__I639 | Main - I63.9 cerebral infarction, unspecified | 0.14 | 0.26 | 0.12 | 0.31 | 0.20 | 0.21 |
| x20015 | Sitting height | 0.22 | 0.13 | 0.16 | 0.34 | 0.18 | 0.21 |
| x41204__J22 | Sec. - J22 unspecified acute lower respiratory infection | 0.23 | 0.26 | 0.13 | 0.21 | 0.20 | 0.20 |
| x41204__G309 | Sec. - G30.9 Alzheimer's disease, unspecified | - | 0.42 | 0.17 | 0.20 | 0.23 | 0.20 |
| x2345 | Ever had bowel cancer screening | 0.18 | 0.21 | 0.18 | 0.23 | 0.22 | 0.20 |
| x41202__J181 | Main - J18.1 lobar pneumonia, unspecified | 0.21 | 0.14 | 0.21 | 0.25 | 0.18 | 0.20 |
| x2267 | Use of sun/UV protection | 0.24 | 0.13 | 0.23 | 0.19 | 0.21 | 0.20 |
| x2178 | Overall health rating | 0.24 | 0.15 | 0.11 | 0.27 | 0.19 | 0.19 |
| x21000 | Ethnic background | 0.28 | 0.06 | 0.24 | 0.17 | 0.21 | 0.19 |
| x2443 | Diabetes diagnosed by doctor | 0.21 | 0.16 | 0.21 | 0.19 | 0.19 | 0.19 |
| x41204__E871 | Sec. - E87.1 hypo-osmolality and hyponatremia | 0.23 | 0.18 | 0.31 | 0.10 | 0.11 | 0.19 |
| x41202__D649 | Main - D64.9 anemia, unspecified | 0.14 | 0.19 | 0.22 | 0.25 | 0.11 | 0.18 |
| x40012__6 | Tumor behavior - malignant, metastatic site | 0.41 | - | - | 0.45 | 0.05 | 0.18 |
| x41204__R268 | Sec. - R26.8 other abnormalities of gait and mobility | 0.09 | 0.27 | 0.16 | 0.20 | 0.18 | 0.18 |
| x728 | Number of vehicles in household | 0.14 | 0.21 | 0.20 | 0.17 | 0.12 | 0.17 |
| x1717 | Skin color | 0.10 | 0.16 | 0.17 | 0.21 | 0.18 | 0.16 |
| x41204__E86 | Sec. - E86 volume depletion | 0.08 | 0.15 | 0.29 | 0.21 | 0.07 | 0.16 |
| x41202__J22 | Main - J22 unspecified acute lower respiratory infection | 0.16 | 0.10 | 0.10 | 0.31 | 0.11 | 0.16 |
| x41204__C771 | Sec. - C77.1 secondary and unspecified malignant neoplasm of intrathoracic lymph nodes | 0.29 | 0.08 | 0.30 | 0.09 | 0.02 | 0.16 |
| x41204__Z922 | Sec. - Z9.22 personal history of long-term (current) use of other medicaments | 0.18 | 0.15 | 0.14 | 0.14 | 0.16 | 0.15 |
| x41204__K573 | Sec. - K57.3 diverticular disease of large intestine without perforation or abscess | 0.17 | 0.13 | 0.12 | 0.20 | 0.11 | 0.15 |
| x41204__L031 | Sec. - L03.1 cellulitis and acute lymphangitis of other parts of limb | 0.05 | 0.13 | 0.20 | 0.19 | 0.18 | 0.15 |
| x6152__6 | emphysema/chronic bronchitis diagnosed by doctor | 0.14 | 0.09 | 0.24 | 0.08 | 0.15 | 0.14 |
| x1835 | Mother still alive | 0.10 | 0.13 | 0.12 | 0.17 | 0.18 | 0.14 |
| x40011__8500 | Histology of cancer tumor — duct adenocarcinoma | 0.11 | 0.08 | 0.23 | 0.14 | 0.13 | 0.14 |
| x47 | Hand grip strength (right) | - | 0.27 | 0.06 | 0.15 | 0.20 | 0.14 |
| x6164__2 | No physical activity in last 4 weeks | 0.14 | 0.10 | 0.11 | 0.16 | 0.15 | 0.13 |
| x23111 | Leg fat percentage (right) | 0.12 | 0.20 | - | 0.34 | - | 0.13 |
| x2306 | Weight change compared with 1 year ago | 0.11 | 0.15 | 0.08 | 0.15 | 0.15 | 0.13 |
| x41204__R11 | Sec. - R11 nausea and vomiting | 0.08 | 0.17 | 0.09 | 0.12 | 0.15 | 0.12 |
| x1448 | Bread type | 0.06 | 0.10 | 0.17 | 0.15 | 0.12 | 0.12 |
| x41204__Z923 | Sec. - Z92.3 personal history of irradiation | 0.15 | 0.06 | - | 0.27 | 0.10 | 0.12 |
| x41204__D696 | Sec. - D69.6 thrombocytopenia, unspecified | 0.11 | 0.05 | 0.04 | 0.25 | 0.12 | 0.12 |
| x41204__K590 | Sec. - K59.0 constipation | 0.09 | 0.12 | 0.19 | 0.06 | 0.09 | 0.11 |
| x1873 | Number of full brothers | 0.12 | 0.09 | 0.11 | 0.15 | 0.05 | 0.11 |
| x41204__L97 | Sec. - L97 non-pressure chronic ulcer of lower limb, not elsewhere classified | 0.18 | 0.11 | 0.09 | - | 0.14 | 0.11 |
| x6145__1 | Serious illness, injury, or assault to yourself in last 2 years | 0.10 | 0.10 | 0.11 | 0.11 | 0.11 | 0.11 |
| x41202__R69 | Main - R69 unknown and unspecified causes of morbidity | 0.10 | 0.05 | 0.09 | 0.17 | 0.12 | 0.10 |
| x2188 | Long-standing illness, disability, or infirmity | 0.10 | 0.12 | 0.14 | 0.08 | 0.08 | 0.10 |
| x6150__100 | No vascular/heart problems diagnosed by doctor | 0.11 | 0.10 | 0.09 | 0.12 | 0.10 | 0.10 |
| x2000 | Worry too long after embarrassment | 0.09 | 0.09 | 0.11 | 0.11 | 0.11 | 0.10 |
| x40006__C343 | C34.3 - malignant neoplasm of lower lobe, bronchus, or lung | 0.07 | 0.09 | 0.10 | - | 0.24 | 0.10 |
| x41204__Z904 | Sec. - Z904 acquired absence of other specified parts of digestive tract | 0.04 | 0.04 | 0.21 | 0.10 | 0.09 | 0.09 |
| x52 | Month of birth | 0.10 | 0.08 | 0.09 | 0.12 | 0.07 | 0.09 |
| x6162__1 | Types of transport used (excluding work) — car/motor vehicle | 0.10 | 0.06 | 0.08 | 0.10 | 0.10 | 0.09 |
| x6144__5 | Eat eggs, diary, wheat, sugar | 0.09 | 0.05 | 0.11 | 0.09 | 0.12 | 0.09 |
| x6164__5 | No heavy DIY physical activity in last 4 weeks | 0.06 | 0.08 | 0.12 | 0.08 | 0.09 | 0.08 |
| x41204__Z871 | Sec. - Z87.1 personal history of diseases of the digestive system | 0.09 | 0.09 | 0.11 | 0.08 | 0.05 | 0.08 |
| x6159__7 | Pain type(s) experienced in last month — knee pain | 0.08 | 0.06 | 0.12 | 0.08 | 0.07 | 0.08 |
| x41204__R13 | Sec. - R13 aphagia and dysphagia | - | 0.02 | 0.23 | 0.12 | 0.03 | 0.08 |
| x41204__Z993 | Sec. - Z99.3 dependence on wheelchair | 0.26 | 0.10 | - | - | 0.02 | 0.08 |
| x41202__K635 | Main - K63.5 polyp of colon | 0.12 | 0.07 | 0.010 | 0.07 | 0.11 | 0.08 |
| x2473 | Other serious medical condition/disability diagnosed by doctor | 0.08 | 0.09 | 0.06 | 0.08 | 0.07 | 0.08 |
| x2207 | Wears glasses or contact lenses | 0.15 | 0.06 | 0.06 | 0.06 | 0.05 | 0.08 |
| x41202__C786 | Main - mesothelioma of pleura | - | 0.24 | 0.13 | - | - | 0.08 |
| x41202__R073 | Main - R07.3 other chest pain | 0.04 | - | 0.09 | 0.09 | 0.15 | 0.07 |
| x41204__M179 | Sec. - M17.9 gonarthrosis, unspecified | 0.06 | 0.04 | 0.13 | 0.08 | 0.06 | 0.07 |
| x41204__R600 | Sec. - R600 peripheral edema | - | 0.05 | - | 0.17 | 0.14 | 0.07 |
| x41204__I252 | Sec. - I25.2 old myocardial infarction | 0.05 | 0.04 | 0.09 | 0.11 | 0.07 | 0.07 |
| x41204__J960 | Sec. - J96.0 acute respiratory failure | - | 0.07 | 0.04 | - | 0.26 | 0.07 |
| x3088 | Contra-indications for spirometry | 0.08 | 0.10 | 0.05 | 0.07 | 0.07 | 0.07 |
| x6159__1 | Pain type(s) experienced in last month — headache | 0.06 | 0.06 | 0.08 | 0.09 | 0.08 | 0.07 |
| x41202__C159 | Main - C15.9 malignant neoplasm of esophagus, unspecified | 0.10 | 0.14 | - | - | 0.12 | 0.07 |
| x6154__3 | Medication for pain relief, constipation, heartburn — paracetamol | 0.08 | 0.09 | 0.10 | 0.02 | 0.07 | 0.07 |
| x41204__K219 | Sec. - K21.9 gastro-esophageal reflux disease without esophagitis | 0.03 | 0.06 | 0.09 | 0.13 | 0.04 | 0.07 |
| x1309 | Fresh fruit intake | 0.07 | 0.08 | - | 0.14 | 0.06 | 0.07 |
| x2296 | Falls in the last year | 0.06 | 0.09 | 0.11 | 0.02 | 0.07 | 0.07 |
| x41202__R074 | Main - R07.4 chest pain, unspecified | 0.05 | 0.12 | 0.09 | 0.04 | 0.05 | 0.07 |
| x41202__R060 | Main - R06.0 dyspnea | 0.09 | - | - | 0.09 | 0.17 | 0.07 |
| x41202__C250 | Main - C25.0 malignant neoplasm of head of pancreas | - | - | 0.10 | 0.16 | 0.08 | 0.07 |
| x6142__4 | Unable to work because of sickness or disability | 0.11 | 0.08 | 0.10 | - | 0.05 | 0.07 |
| x41202__C443 | Main - C44.3 skin of other and unspecified parts of face | 0.05 | 0.006 | 0.09 | 0.08 | 0.09 | 0.06 |
| x2492 | Taking other prescription medications | 0.04 | 0.05 | 0.07 | 0.04 | 0.10 | 0.06 |
| x6139__1 | A gas hob or gas cooker for cooking/heating | 0.05 | 0.07 | 0.05 | 0.07 | 0.05 | 0.06 |
| x41202__K409 | Main - K40.9 unilateral or unspecified inguinal hernia | 0.04 | 0.09 | 0.02 | 0.07 | 0.07 | 0.06 |
| x41204__I739 | Sec. - I73.9 peripheral vascular disease, unspecified | 0.05 | 0.08 | 0.16 | - | - | 0.06 |
| x41204__Z512 | Sec. - Z51.2 other chemotherapy | 0.07 | 0.06 | 0.08 | 0.07 | - | 0.06 |
| x41202__R31 | Main - R31 hematuria | 0.04 | - | 0.06 | 0.06 | 0.12 | 0.05 |
| x6164__4 | Light DIY physical activity in last 4 weeks | 0.06 | 0.03 | 0.07 | 0.06 | 0.05 | 0.05 |
| x41204__E669 | Sec. - E66.9 obesity | 0.03 | 0.14 | 0.08 | - | 0.006 | 0.05 |
| x1883 | Number of full sisters | 0.05 | 0.05 | 0.07 | 0.05 | 0.03 | 0.05 |
| x41202__C795 | Main - C79.5 sec. malig. neoplasm of bone and bone marrow | 0.15 | 0.05 | - | 0.06 | - | 0.05 |
| x41204__Y836 | Sec. - Y83.6 removal of other organ (partial) (total) as the cause of abnormal reaction of the patient, or of later complication | 0.01 | 0.06 | - | 0.09 | 0.08 | 0.05 |
| x1359 | Poultry intake | - | 0.07 | 0.09 | - | 0.09 | 0.05 |
| x41204__N19 | Sec. - N19 unspecified kidney failure | 0.02 | 0.11 | 0.06 | 0.05 | - | 0.05 |
| x41204__Z864 | Sec. - Z86.4 personal history of psychoactive substance abuse | 0.07 | 0.07 | 0.05 | 0.05 | 0.002 | 0.05 |
| x6149__100 | No mouth/teeth dental problems | 0.08 | 0.05 | 0.06 | 0.03 | 0.02 | 0.05 |
| x41204__C772 | Sec. - C77.2 secondary and unspecified malignant neoplasm of intra-abdominal lymph nodes. | 0.08 | 0.08 | 0.01 | - | 0.07 | 0.05 |
| x41204__Z538 | Sec. - Z53.8 procedure and treatment not carried out for other reasons | 0.02 | 0.05 | 0.06 | 0.003 | 0.09 | 0.05 |
| x864 | Number of days/week walked 10+ minutes | 0.04 | 0.06 | 0.02 | 0.04 | 0.07 | 0.05 |
| x41202__A419 | Main - A41.9 sepsis, unspecified organism | 0.01 | 0.02 | 0.07 | 0.02 | 0.10 | 0.05 |
| x41202__K573 | Main - K57.3 diverticular disease of large intestine without perforation or abscess | 0.03 | - | 0.07 | 0.09 | 0.04 | 0.05 |
| x2316 | Wheeze or whistling in the chest in last year | 0.04 | 0.07 | 0.04 | 0.06 | 0.02 | 0.04 |
| x2257 | Hearing difficulty/problems with background noise | 0.06 | 0.03 | 0.04 | 0.04 | 0.04 | 0.04 |
| x41202__M169 | Main - M16.9 coxarthrosis, unspecified | 0.03 | - | 0.08 | 0.010 | 0.09 | 0.04 |
| x1289 | Cooked vegetable intake | 0.07 | 0.01 | 0.03 | 0.09 | 0.02 | 0.04 |
| x41204__F329 | Sec. - F32.9 depressive episode, unspecified | 0.02 | 0.02 | - | 0.11 | 0.05 | 0.04 |
| x41204__F171 | Sec. - F17.1 mental and behavioral disorders due to use of tobacco, harmful use | 0.03 | 0.01 | 0.06 | 0.01 | 0.09 | 0.04 |
| x6152__9 | Hay fever, allergic rhinitis or eczema diagnosed by doctor | 0.03 | 0.07 | 0.06 | 0.04 | - | 0.04 |
| x41202__C920 | Main - C92.0 acute myeloblastic leukemia | - | - | - | 0.20 | - | 0.04 |
| x6164__1 | Types of physical activity in last 4 weeks — walking for pleasure | 0.07 | 0.06 | 0.02 | 0.03 | 0.007 | 0.04 |
| x41202__R410 | Main - R41.0 disorientation, unspecified | - | - | - | 0.04 | 0.14 | 0.04 |
| x6154__1 | Medication for pain relief, constipation, heartburn — aspirin | 0.03 | 0.02 | - | 0.06 | 0.07 | 0.04 |
| x6149__6 | Mouth/teeth dental problems — dentures | 0.009 | 0.04 | 0.05 | 0.05 | 0.04 | 0.04 |
| x41204__Z874 | Sec. - Z87.4 personal history of diseases of genitourinary system | 0.06 | - | 0.04 | 0.06 | 0.02 | 0.03 |
| x41204__E780 | Sec. - E78.0 pure hypercholesterolemia | 0.03 | 0.05 | 0.06 | 0.01 | 0.01 | 0.03 |
| x41204__I251 | Sec. - I25.1 atherosclerotic heart disease of native coronary artery | 0.06 | 0.06 | 0.05 | - | - | 0.03 |
| x41204__W199 | Sec. - W19.9 unspecified fall home during unspecified activity | 0.06 | - | - | - | 0.10 | 0.03 |
| x1349 | Processed meat intake | - | 0.08 | 0.08 | - | - | 0.03 |
| x1960 | Fed-up feelings | 0.04 | 0.01 | 0.04 | 0.03 | 0.03 | 0.03 |
| x41204__Z966 | Sec. - Z96.6 presence of orthopedic joint implants | 0.05 | - | 0.07 | 0.03 | 0.004 | 0.03 |
| x6164__3 | Types of physical activity in last 4 weeks — no strenuous sports | 0.08 | - | 0.04 | - | 0.03 | 0.03 |
| x40012__1 | Tumor behavior - uncertain whether benign or malignant | - | 0.08 | - | 0.02 | 0.04 | 0.03 |
| x2463 | Fractured/broken bones in last 5 years | 0.07 | - | - | - | 0.06 | 0.03 |
| x41204__K529 | Sec. - K52.9 noninfective gastroenteritis and colitis, unspecified | 0.04 | 0.02 | 0.06 | - | - | 0.03 |
| x6159__3 | Pain type(s) experienced in last month — neck or shoulder pain | 0.03 | 0.02 | 0.01 | 0.03 | 0.03 | 0.03 |
| x6145__3 | Death of a close relative in last 2 years | - | 0.05 | 0.02 | 0.05 | - | 0.03 |
| x41204__K703 | Sec. - K70.3 alcoholic cirrhosis of liver | 0.12 | - | - | - | - | 0.02 |
| x6145__2 | Serious illness, injury, or assault of a close relative in last 2 years | 0.07 | - | - | 0.006 | 0.04 | 0.02 |
| x41204__R91 | Sec. - R91 abnormal findings on diagnostic imaging of lung | - | 0.11 | - | - | - | 0.02 |
| x41204__Z968 | Sec. - Z96.8 presence of other specified functional implants | 0.05 | - | - | - | 0.06 | 0.02 |
| x41202__J440 | Main - J44.0 chronic obstructive pulmonary disease with acute lower respiratory infection. | - | - | - | - | 0.11 | 0.02 |
| x41202__K831 | Main - K83.1 obstruction of bile duct | 0.11 | - | - | - | - | 0.02 |
| x41202__C787 | Main - secondary malignant neoplasm of liver and intrahepatic bile duct. | - | - | 0.07 | 0.03 | - | 0.02 |
| x41204__I517 | Sec. - I51.7 cardiomegaly | 0.10 | - | - | 0.003 | - | 0.02 |
| x40006__C259 | C25.9 - malignant neoplasm of pancreas, unspecified. | - | - | - | - | 0.10 | 0.02 |
| x6138__6 | Qualifications — other professional qualifications | 0.02 | 0.03 | 0.02 | 0.03 | - | 0.02 |
| x41202__K219 | Main - K21.9 gastro-esophageal reflux disease without esophagitis | 0.09 | - | - | - | - | 0.02 |
| x2040 | Risk taking | 0.03 | 0.04 | - | 0.01 | 0.007 | 0.02 |
| x6154__6 | Medication for pain relief, constipation, heartburn — laxatives | 0.07 | - | 0.01 | - | - | 0.02 |
| x41204__G409 | Sec. - G40.9 epilepsy, unspecified | - | - | 0.08 | - | - | 0.02 |
| x41204__C61 | Sec. - C61 malignant neoplasm of prostate | - | 0.006 | 0.007 | - | 0.07 | 0.02 |
| x41204__Z824 | Sec. - Z82.4 family history of ischemic heart disease and other diseases of the circulatory system | 0.03 | 0.008 | - | 0.05 | - | 0.02 |
| x6179__2 | Mineral and other dietary supplements — glucosamine | 0.04 | 0.04 | - | 0.00001 | - | 0.02 |
| x41204__K766 | Sec. - K76.6 portal hypertension | - | - | - | 0.08 | - | 0.02 |
| x1797 | Father still alive | 0.04 | - | 0.02 | 0.02 | - | 0.02 |
| x41204__C770 | Sec. - C77.0 secondary and unspecified malignant neoplasm of lymph nodes of head, face, and neck | - | - | - | 0.07 | - | 0.01 |
| x41204__Z888 | Sec. - Z88.8 allergy status to other drugs, medicaments, and biological substances | - | 0.04 | 0.03 | - | - | 0.01 |
| x6155__100 | Vitamin and mineral supplements — none | 0.002 | 0.04 | 0.008 | 0.02 | - | 0.01 |
| x41204__N40 | Sec. - N40 enlarged prostate | 0.01 | - | - | 0.02 | 0.04 | 0.01 |
| x41204__R000 | Sec. - R00.00 tachycardia, unspecified | - | 0.07 | - | - | - | 0.01 |
| x41204__N183 | Sec. - N18.3 chronic kidney disease, stage 3 (moderate) | - | 0.06 | 0.009 | - | - | 0.01 |
| x2030 | Guilty feelings | 0.03 | - | 0.02 | 0.02 | - | 0.01 |
| x1428 | Spread type | - | 0.03 | - | 0.008 | 0.03 | 0.01 |
| x6159__6 | Pain type(s) experienced in last month — hip pain | - | 0.05 | - | 0.01 | - | 0.01 |
| x6142__2 | Current employment status - retired | 0.01 | - | - | 0.003 | 0.05 | 0.01 |
| x41202__R104 | Main - R10.4 generalized abdominal pain | - | 0.06 | - | - | - | 0.01 |
| x6139__3 | Not using an open solid fuel fire regularly in winter time | 0.05 | 0.008 | - | - | - | 0.01 |
| x6179__1 | Mineral and other dietary supplements — fish oil (including cod liver oil) | 0.02 | 0.02 | - | 0.008 | 0.002 | 0.01 |
| x1747 | Hair color (natural, before greying) | - | - | 0.05 | - | - | 0.01 |
| x41204__R55 | Sec. - R55 syncope and collapse | - | - | - | - | 0.05 | 0.010 |
| x6155__6 | Vitamin and mineral supplements — folic acid or Folate | - | - | - | 0.05 | - | 0.009 |
| x6154__100 | No medication for pain relief, constipation, heartburn | - | - | 0.05 | - | - | 0.009 |
| x41204__J459 | Sec. - J45.9 other and unspecified asthma | 0.03 | - | - | 0.01 | - | 0.009 |
| x6159__4 | Pain type(s) experienced in last month — back pain | - | 0.006 | 0.01 | 0.02 | - | 0.009 |
| x41204__R509 | Sec. - R50.9 fever, unspecified | - | - | - | 0.04 | - | 0.009 |
| x41204__N189 | Sec. - N18.9 chronic kidney disease, unspecified | - | 0.04 | - | - | - | 0.009 |
| x41201__Y831 | Main - Y83.1 surgical operation with implant of artificial internal device | - | - | - | 0.04 | - | 0.008 |
| x41202__H269 | Main - H26.9 unspecified cataract | - | - | - | 0.03 | 0.01 | 0.008 |
| x41204__M8199 | Sec. - M81.99 osteoporosis, unspecified, site unspecified | - | 0.04 | - | - | - | 0.008 |
| x6139__2 | A gas fire that you use regularly in winter time | 0.02 | - | 0.01 | - | 0.005 | 0.008 |
| x41204__Z961 | Sec. - Z96.1 presence of intraocular lens | - | - | - | - | 0.04 | 0.008 |
| x41204__K449 | Sec. - K44.9 diaphragmatic hernia without obstruction or gangrene | 0.04 | - | - | - | - | 0.008 |
| x41204__R53 | Sec. - R53 malaise and fatigue | - | 0.03 | - | - | - | 0.007 |
| x41202__N40 | Main - N40 enlarged prostate | - | 0.03 | - | - | - | 0.007 |
| x136 | Number of operations, self-reported | - | 0.01 | - | 0.02 | - | 0.007 |
| x6149__1 | Mouth/teeth dental problems - mouth ulcers | - | - | - | - | 0.03 | 0.007 |
| x2100 | Seen a psychiatrist for nerves, anxiety, tension, or depression | - | 0.02 | 0.01 | 0.002 | - | 0.007 |
| x41202__C450 | Main - C45.0 | - | - | 0.03 | - | - | 0.007 |
| x41202__I219 | Main - I21.9 acute myocardial infarction, unspecified | 0.03 | - | - | - | - | 0.006 |
| x6144__4 | Never eat sugar or foods/drinks containing sugar | 0.02 | - | - | - | 0.01 | 0.006 |
| x1488 | Tea intake | 0.004 | 0.0000002 | - | - | 0.03 | 0.006 |
| x1548 | Variation in diet | - | - | - | - | 0.03 | 0.006 |
| x2060 | Frequency of unenthusiasm / disinterest in last 2 weeks | - | 0.03 | - | - | - | 0.006 |
| x41204__Z907 | Sec. - Z907 acquired absence of genital organ(s) | 0.03 | - | - | - | - | 0.005 |
| x41202__I251 | Main - I25.1 atherosclerotic heart disease of native coronary artery | - | - | - | 0.03 | - | 0.005 |
| x6138__4 | Qualifications — CSEs or equivalent | 0.03 | - | - | - | - | 0.005 |
| x41202__Z087 | Main - Z08.7 follow-up examination after combined treatment for malignant neoplasm | - | - | - | 0.02 | - | 0.005 |
| x41202__R91 | Main - R91 abnormal findings on diagnostic imaging of lung | - | - | - | - | 0.02 | 0.004 |
| x41204__I10 | Sec. - I10 essential (primary) hypertension | - | - | - | - | 0.02 | 0.004 |
| x1950 | Sensitivity / hurt feelings | 0.007 | - | - | - | 0.01 | 0.004 |
| x1369 | Beef intake | - | - | - | 0.02 | - | 0.004 |
| x41202__K802 | Main - K80.2 calculus of gallbladder without cholecystitis | - | - | - | 0.02 | - | 0.003 |
| x6155__3 | Vitamin and mineral supplements — vitamin C | - | - | 0.02 | - | - | 0.003 |
| x120 | Birth weight known | - | - | 0.02 | - | - | 0.003 |
| x2010 | Suffer from 'nerves' | - | - | 0.001 | - | 0.01 | 0.003 |
| x6138__100 | Qualifications — none | 0.002 | - | 0.002 | - | 0.010 | 0.003 |
| x41204__K221 | Sec. - K22.1 ulcer of esophagus | - | - | - | 0.01 | - | 0.002 |
| x6149__3 | Mouth/teeth dental problems - bleeding gums | - | - | - | 0.0001 | 0.01 | 0.002 |
| x6152__8 | Asthma diagnosed by doctor | - | - | 0.01 | - | - | 0.002 |
| x6155__7 | Vitamin and mineral supplements — multivitamins +/- minerals | - | 0.01 | - | - | - | 0.002 |
| x6144__1 | Never eat eggs or foods containing eggs | - | - | 0.01 | - | - | 0.002 |
| x1697 | Comparative height size at age 10 | 0.009 | - | - | - | - | 0.002 |
| x41204__K635 | Sec. - K63.5 polyp of colon | - | - | - | 0.009 | - | 0.002 |
| x6155__2 | Vitamin and mineral supplements — vitamin B | - | - | 0.008 | - | - | 0.002 |
| x41202__K297 | Main - K29.7 gastritis, unspecified | - | - | - | 0.008 | - | 0.002 |
| x6162__2 | Types of transport used (excluding work) — walk | 0.007 | - | - | 0.001 | - | 0.002 |
| x6159__5 | Pain type(s) experienced in last month — stomach or abdominal pain | 0.008 | - | - | - | - | 0.002 |
| x1687 | Comparative body size at age 10 | - | 0.007 | - | - | - | 0.001 |
| x1930 | Miserableness | - | 0.007 | - | - | - | 0.001 |
| x2227 | Other eye problems | - | - | - | - | 0.006 | 0.001 |
| x41204__J449 | Sec. - J44.9 chronic obstructive pulmonary disease, unspecified | 0.006 | - | - | - | - | 0.001 |
| x41202__I48 | Main - I48 atrial fibrillation and flutter | - | 0.006 | - | - | - | 0.001 |
| x6154__2 | Medication for pain relief, constipation, heartburn — ibuprofen (e.g. Nurofen) | 0.005 | - | - | - | - | 0.001 |
| x6162__3 | Types of transport used (excluding work) — public transport | 0.005 | - | - | - | - | 0.001 |
| x137 | Number of treatments/medications taken | - | - | 0.005 | - | - | 0.001 |
| x41204__Z720 | Sec. - Z72.0 tobacco use | - | 0.005 | - | - | - | 0.001 |
| x1920 | Mood swings | - | - | 0.003 | - | - | 0.0007 |
| x1980 | Worrier / anxious feelings | 0.003 | - | - | - | - | 0.0006 |
| x41204__E119 | Sec. - E11.9 non-insulin-dependent diabetes mellitus, without complications | - | - | - | - | 0.003 | 0.0005 |
| x1647 | Country of birth (UK/elsewhere) | - | 0.003 | - | - | - | 0.0005 |
| x2090 | Seen doctor (GP) for nerves, anxiety, tension, or depression | 0.002 | - | - | - | - | 0.0003 |
| x1727 | Ease of skin tanning | - | - | 0.001 | - | - | 0.0002 |
| x41202__K30 | Main - K30 functional dyspepsia | - | - | 0.0005 | - | - | 0.0001 |
| x41204__J440 | Sec. - J44.0 chronic obstructive pulmonary disease with acute lower respiratory infection | 0.0004 | - | - | - | - | 0.0001 |

Abbreviations: CSE, certificate of secondary education; NOS, not otherwise specified; Sec., secondary diagnosis.

^a^ International classification of diseases (ICD) codes are given for diagnoses.

.


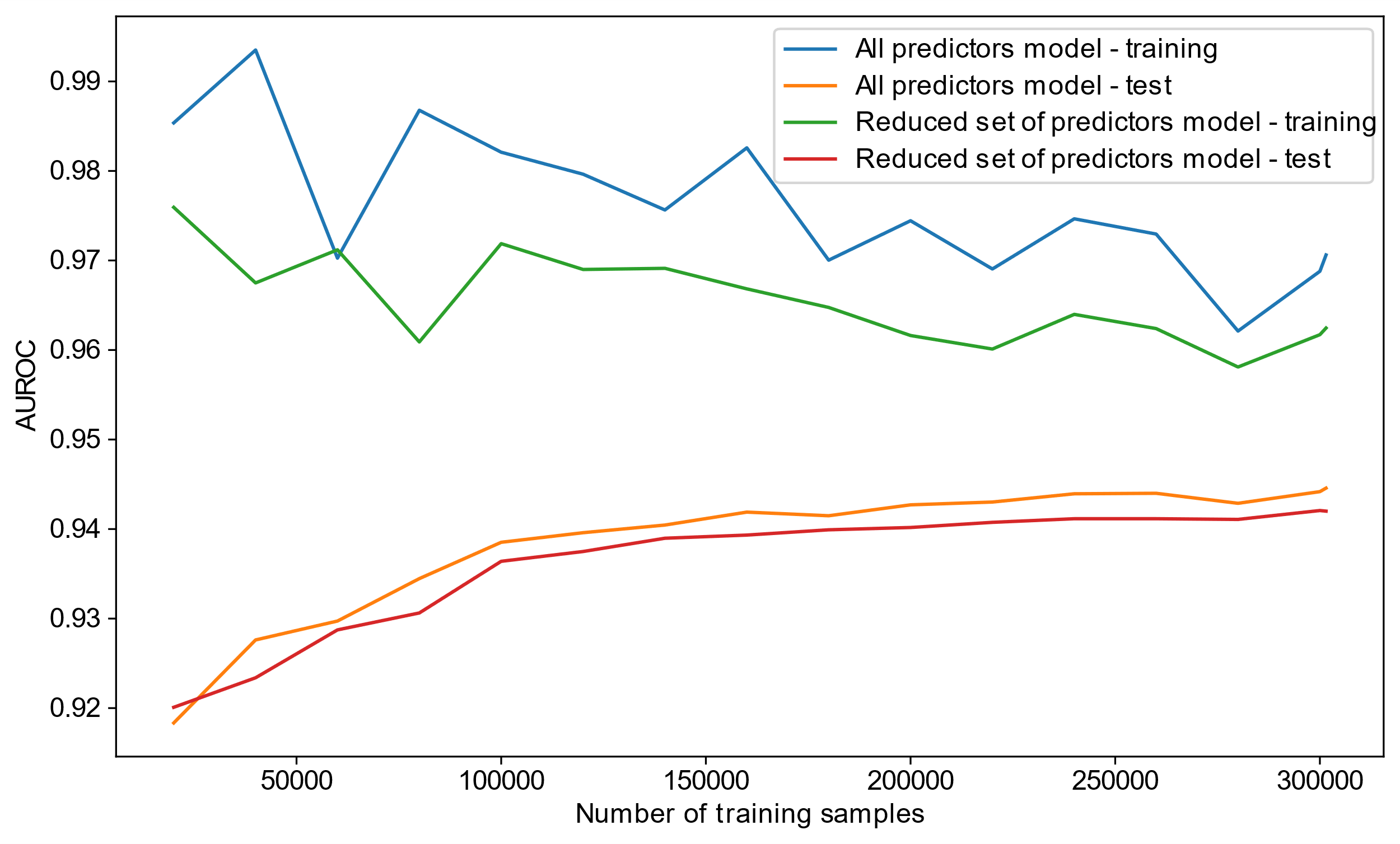


**Supplementary Figure S1**. Learning curve analysis showing performance of set of all predictors model and reduced set of predictors models on training set and test set with increasing number of samples used for training. Model performance was assessed using area under the receiver operating characteristics curve (AUROC).


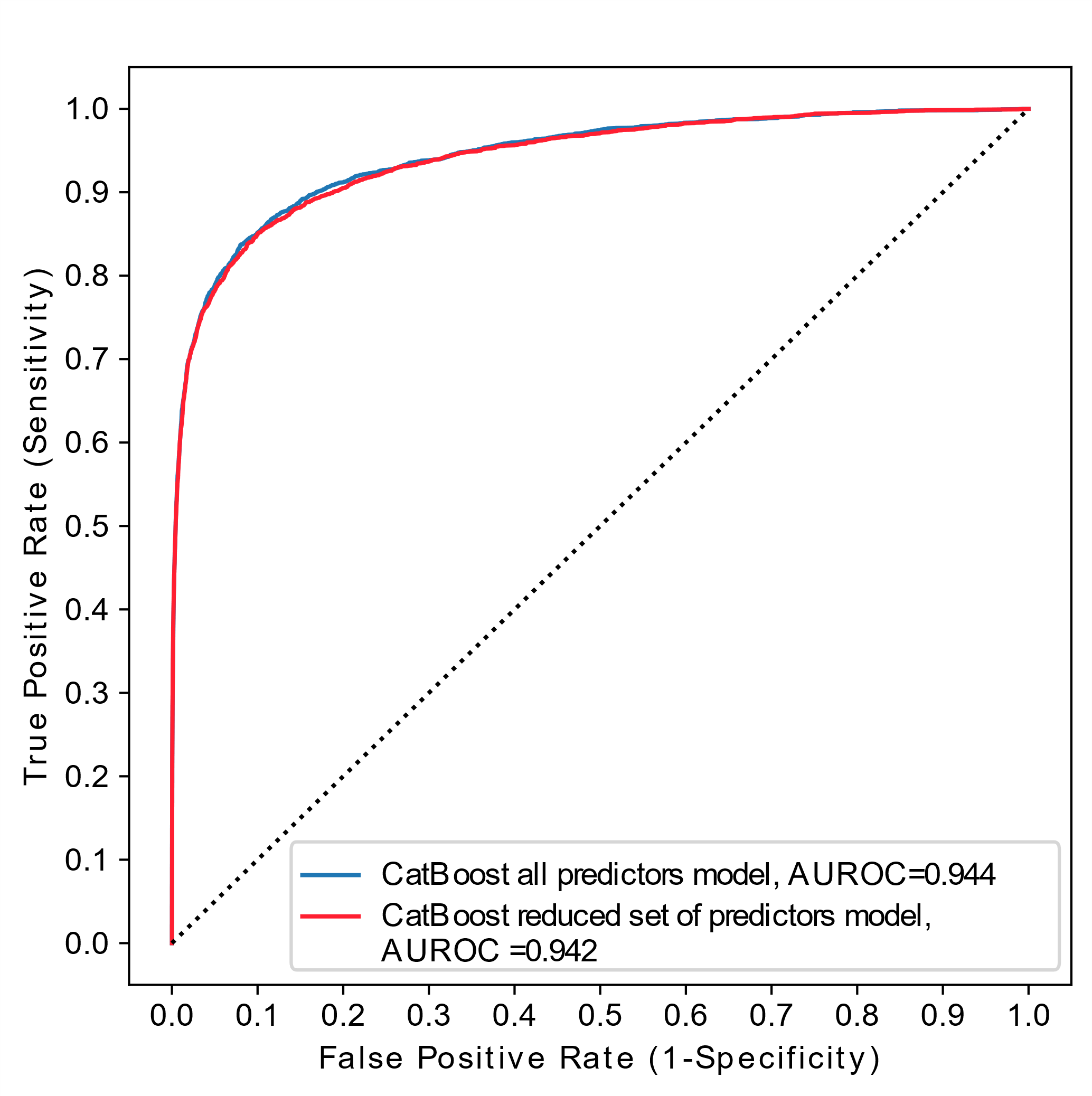


**Supplementary Figure S2**. Receiver operating characteristics (ROC) curves for gradient boosting decision trees (GBDT) models with all predictors and with the 193 important predictors.


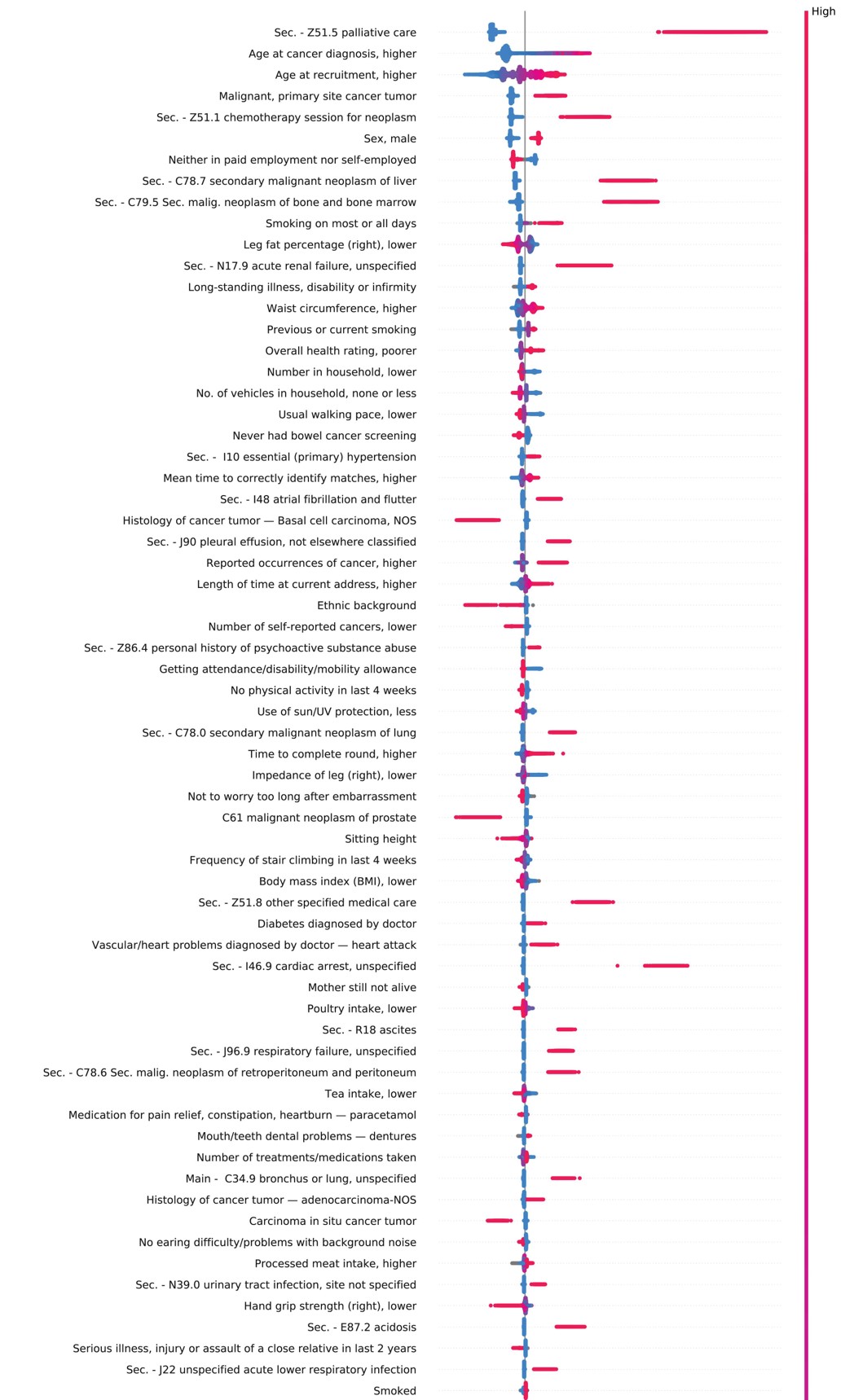


**
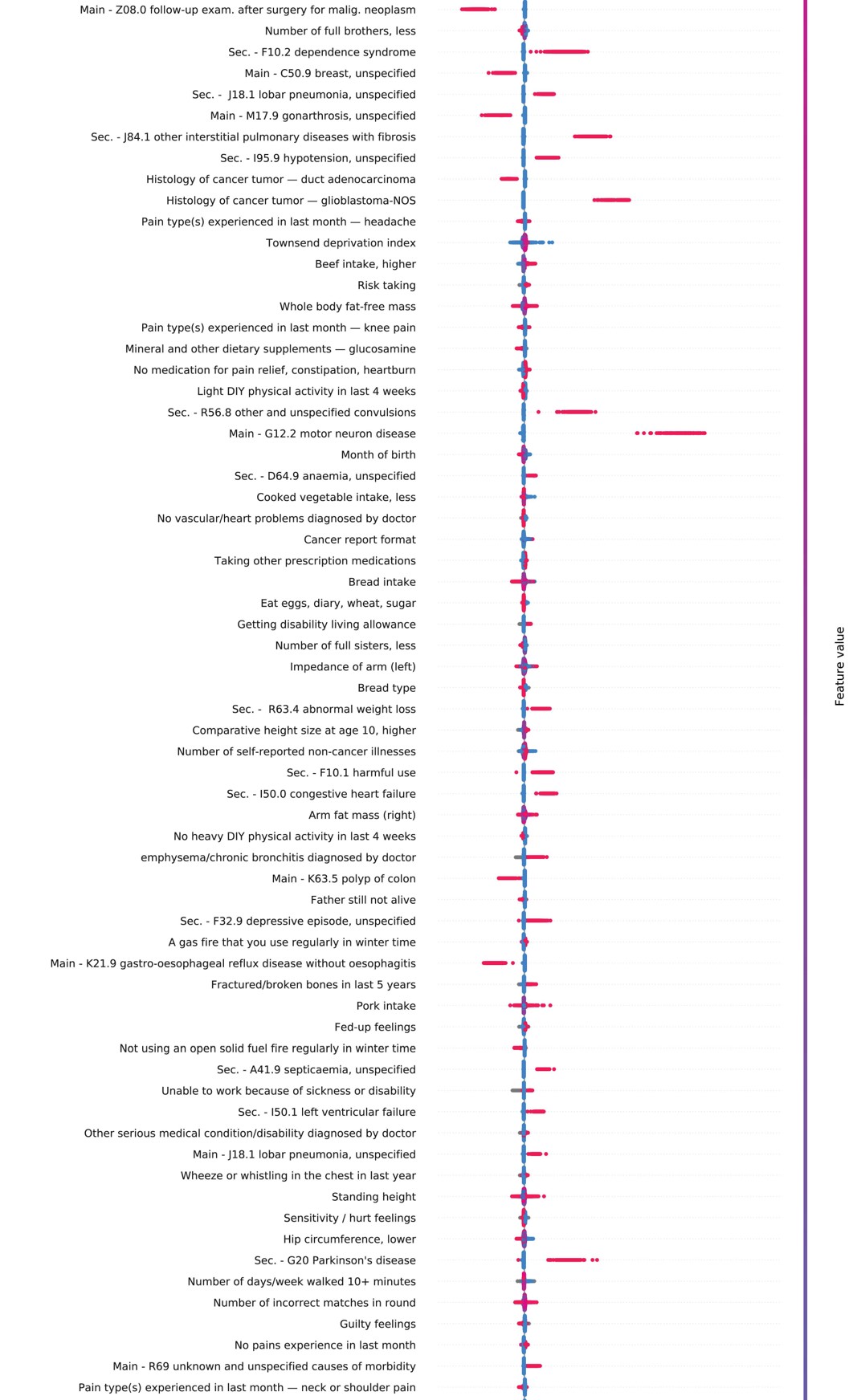
**

**
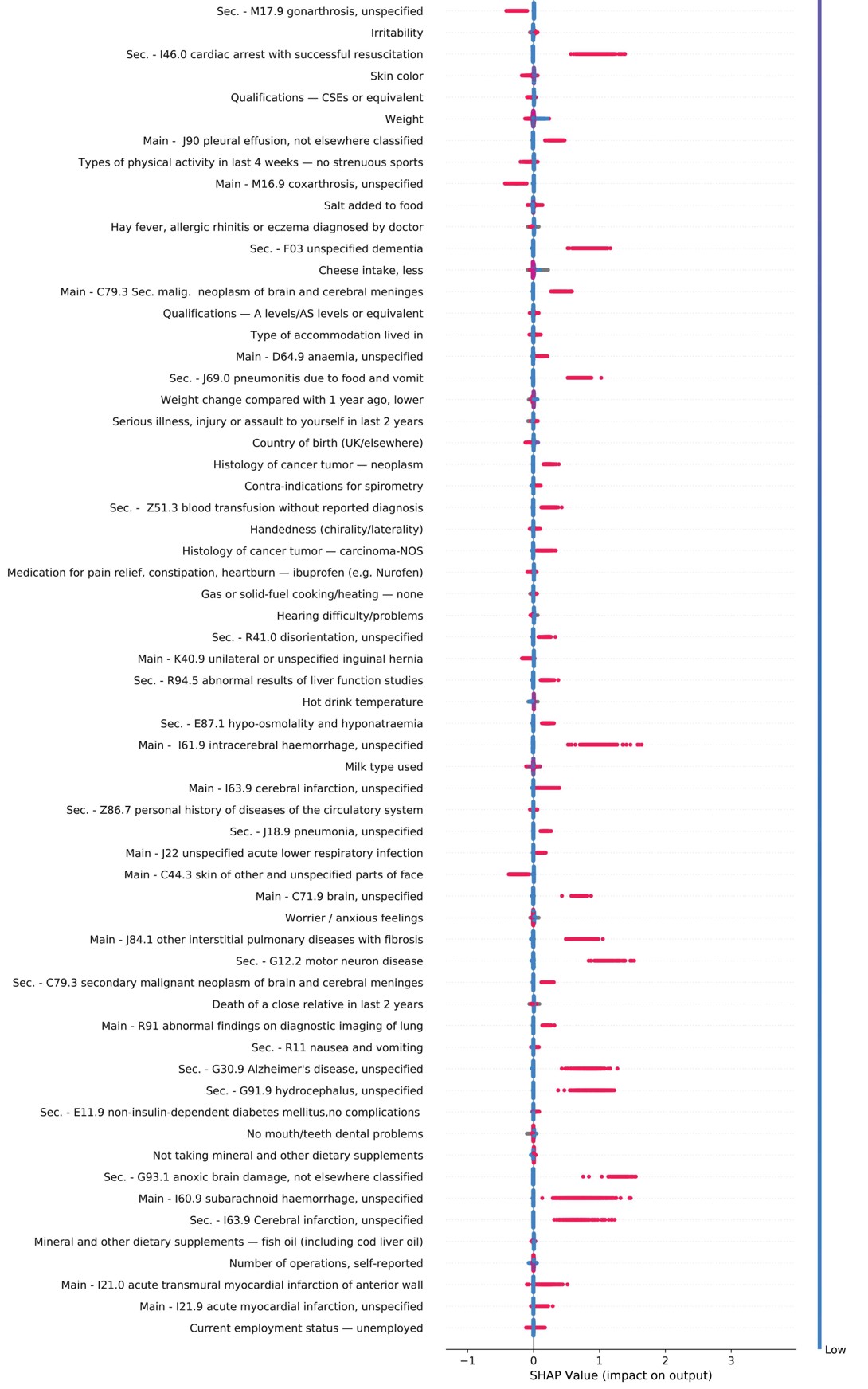
**

**Supplementary Figure S3**. SHAP (SHapley Additive exPlanation) variable importance for the 193 important predictors on all-cause mortality in the UK Biobank. Each point on the plot is a SHAP value for a predictor and a sample. The position on the y-axis is determined by the predictor and on the x-axis by the SHAP value. The colour gradient represents predictor values from low (blue) to high (red). Grey dots represent samples with missing information. International classification of diseases (ICD) codes are given for diagnoses. Predictor names are modified by adding additional text after a ‘—’ for some predictors to reflect how higher value(s) are coded. Abbreviations: A level, advanced level; AS level, advanced subsidiary level; CSE, certificate of secondary education; NOS, not otherwise specified; Sec., secondary diagnosis.

**
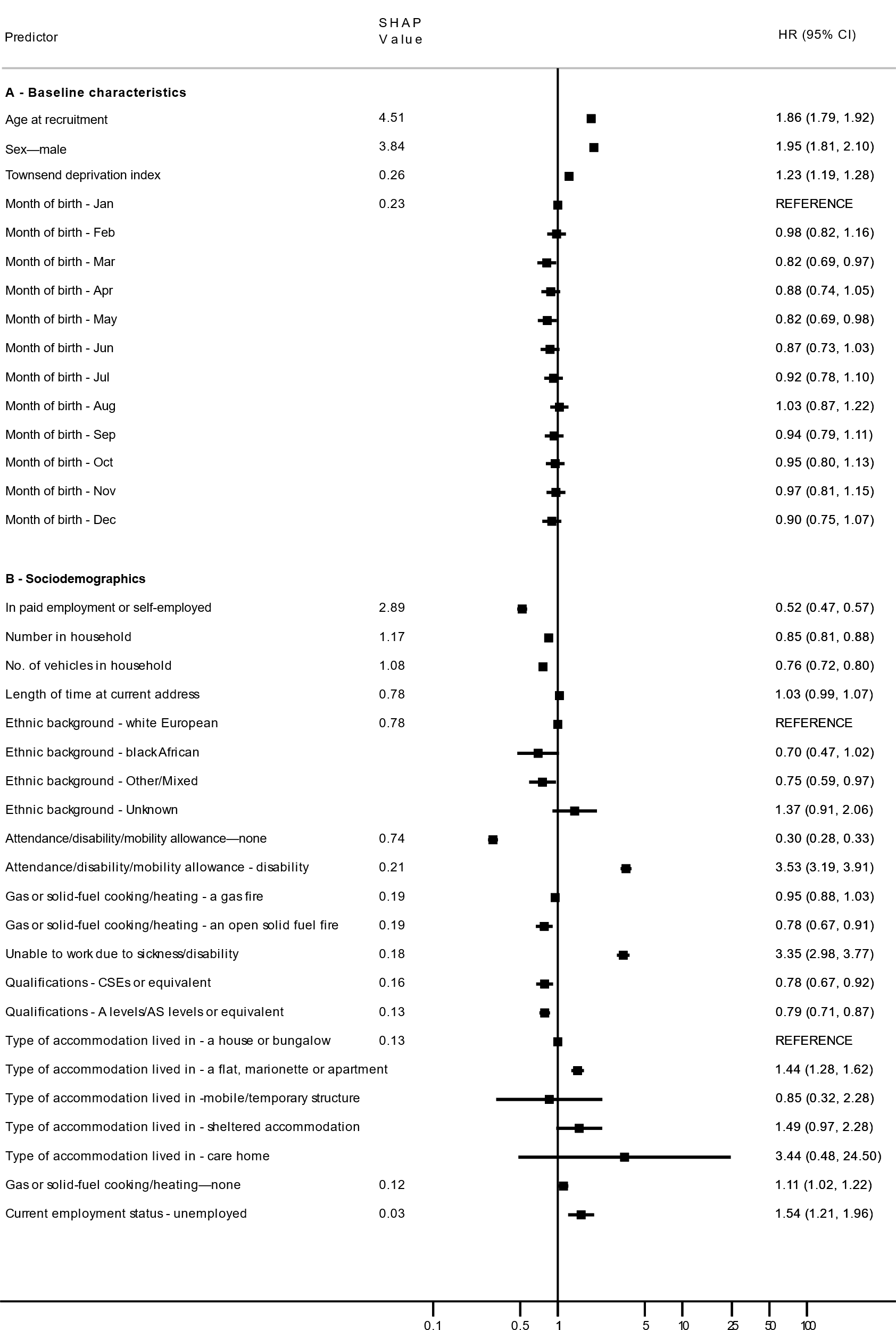
**

**
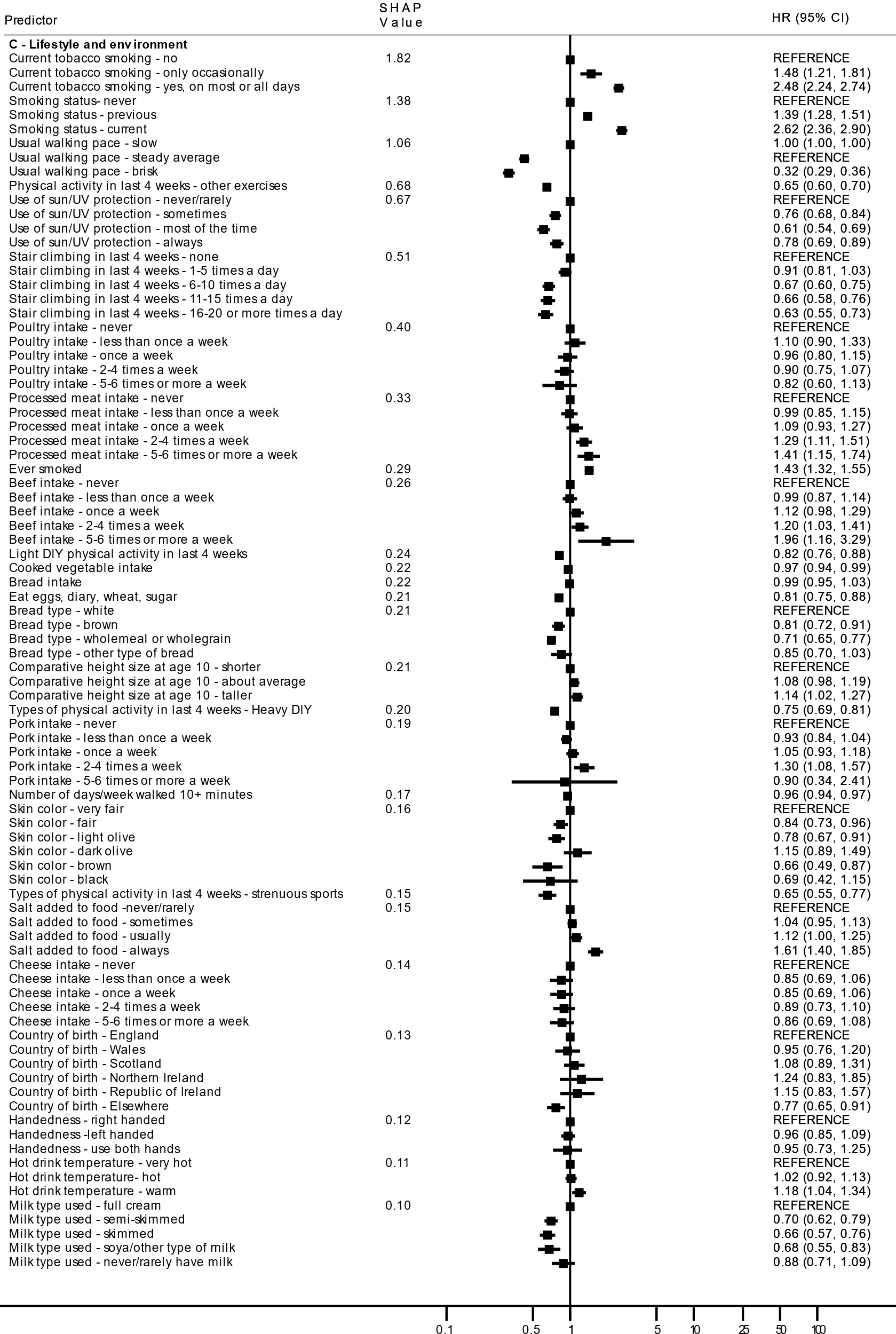
**

**
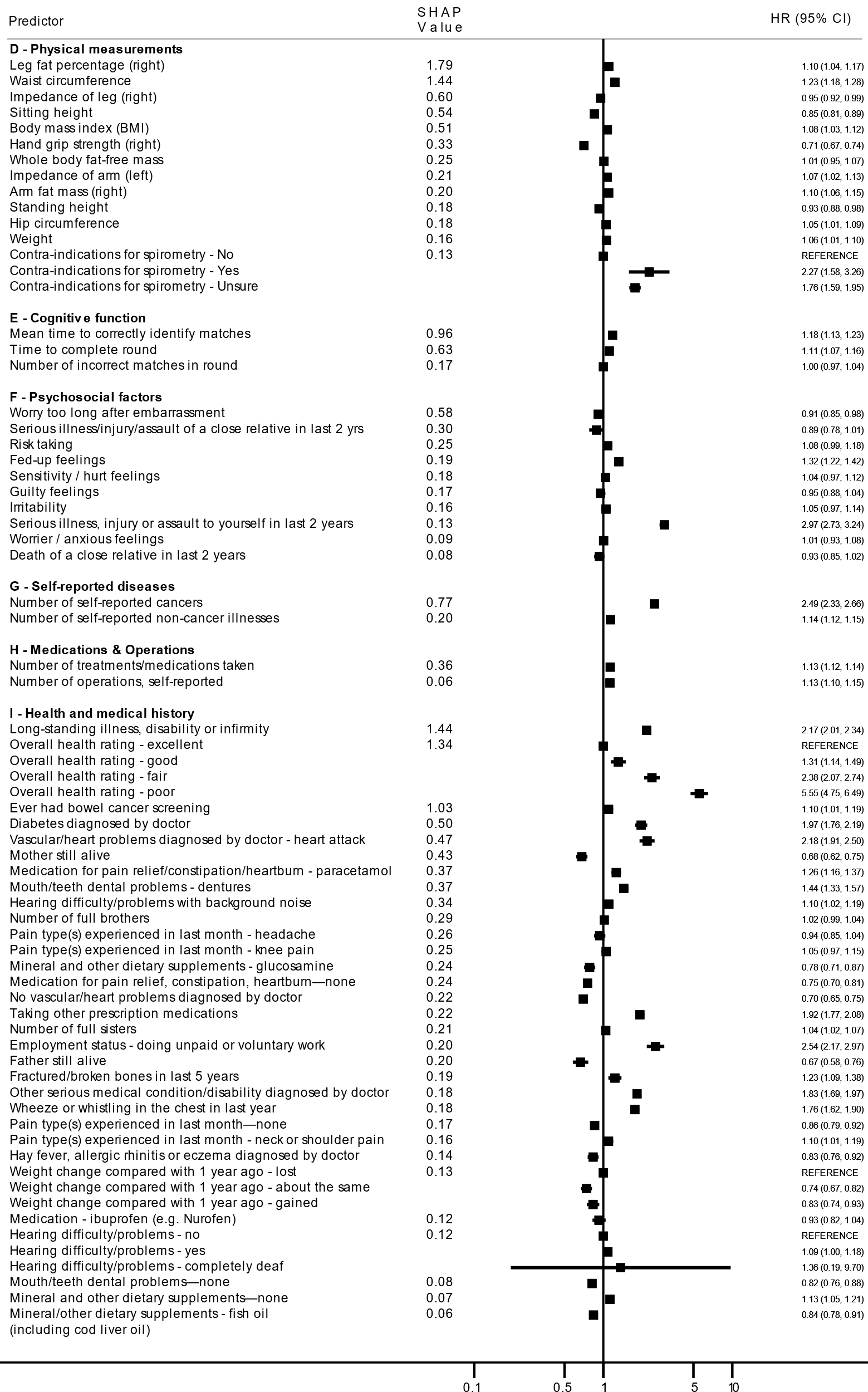
**

**
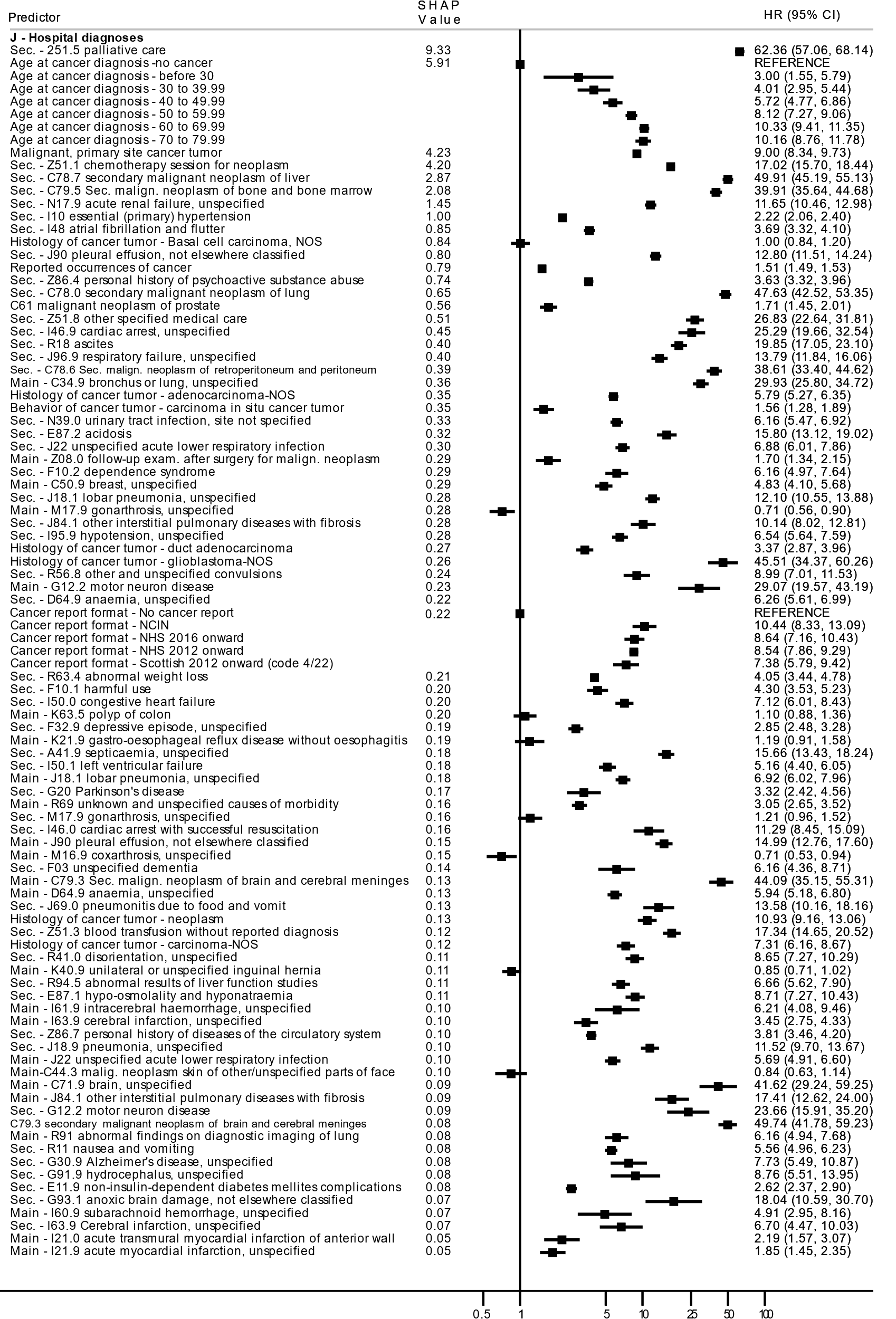
**

**Supplementary Figure S4.** Hazard ratio (HR) with 95% confidence interval and SHAP (SHapley Additive exPlanation) values arranged by predictor category for all the importance predictors (193 predictors). International classification of diseases (ICD) codes are given for diagnoses. Predictor names are modified by adding additional text after a ‘—’ for some predictors to reflect how higher value(s) are coded. Abbreviations: HR, hazard ratio; NOS, not otherwise specified; Sec., secondary diagnosis.


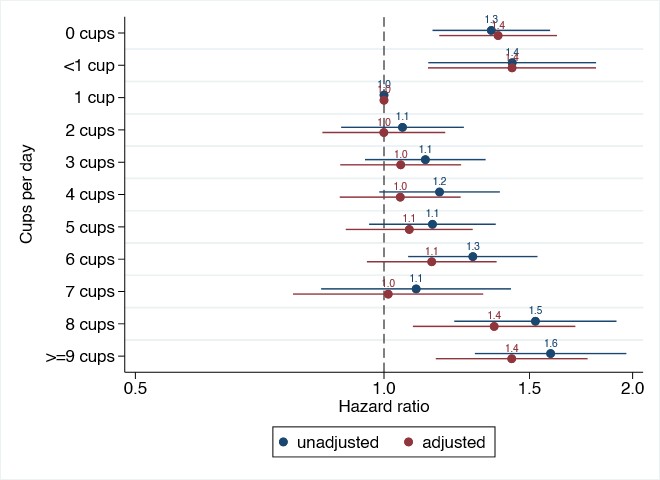


**Supplementary Figure S5.** Association of tea intake with all-cause mortality, unadjusted and adjusted for age, sex, Townsend deprivation index, assessment center and month of birth. Reference group is one cup per day. 95% confidence intervals are shown for hazard ratios using horizontal bars.

#
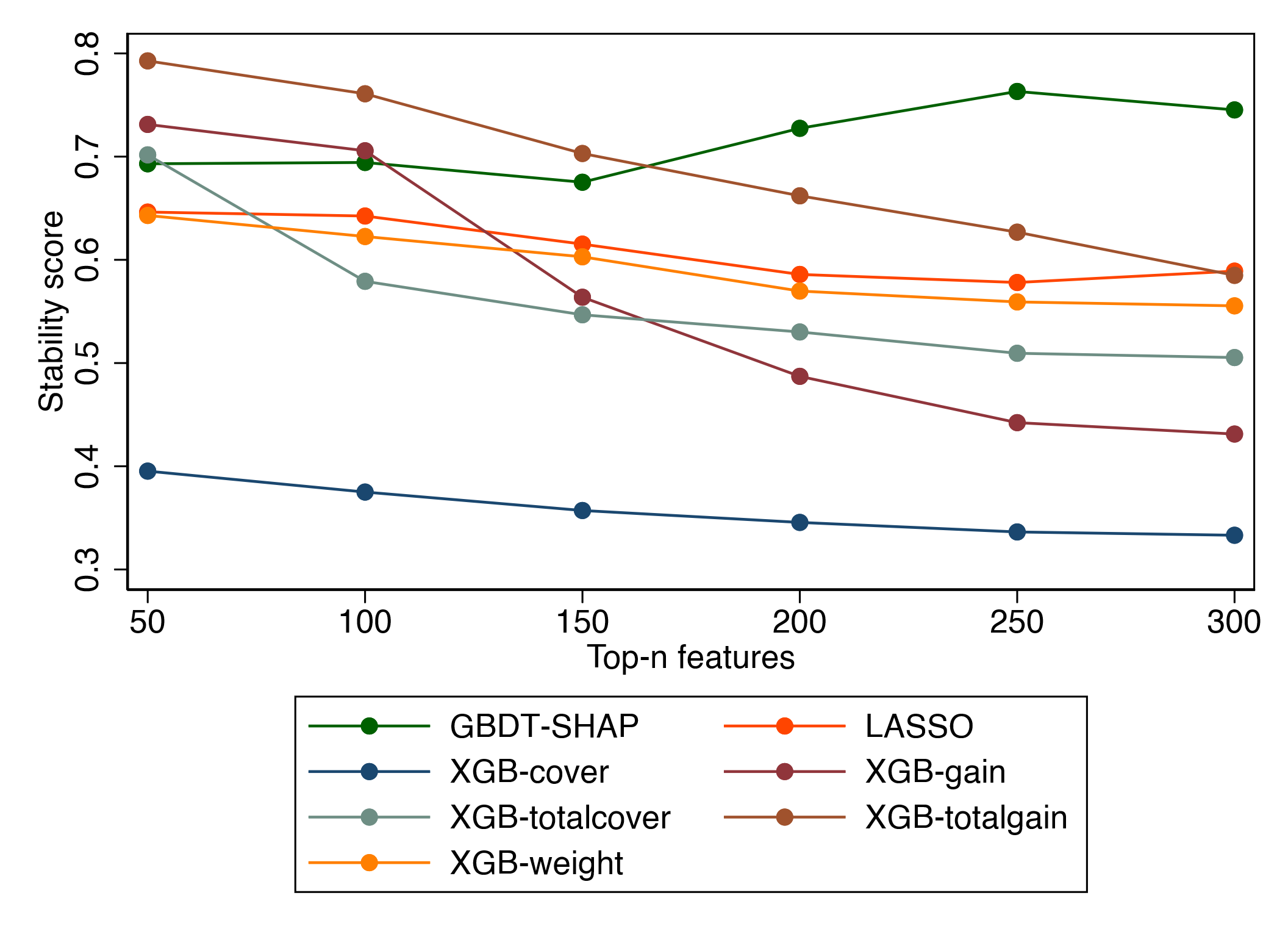


**Supplementary Figure S6.** Unadjusted feature stability score comparison. The figure shows unadjusted stability scores for feature selection methods, CatBoost with SHAP values (GBDT-SHAP), LASSO logistic regression and five different XGBoost (XGB) built-in feature importance calculation methods (weight, gain, cover, total gain, and total cover).


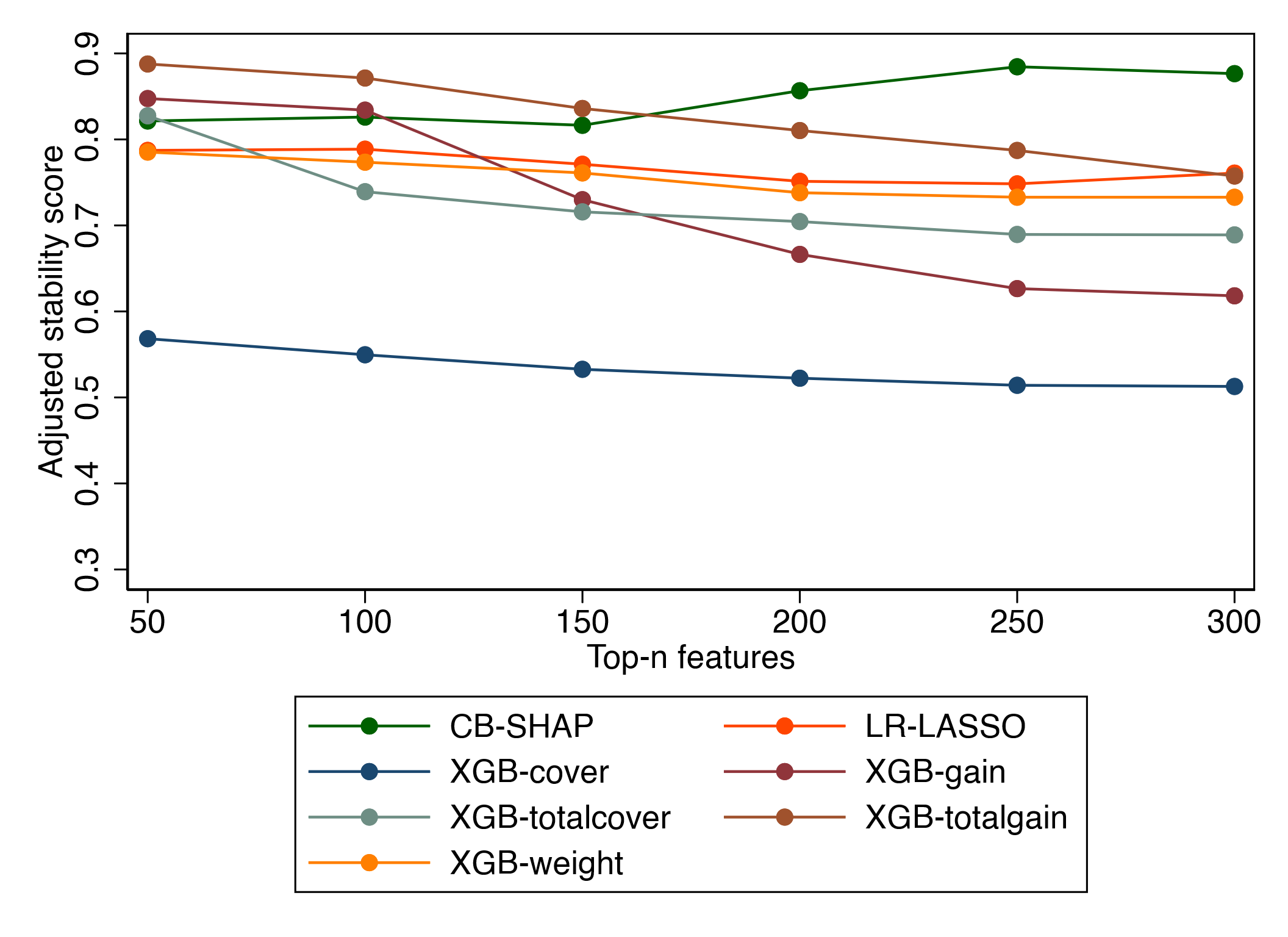


**Supplementary Figure S7**. Adjusted feature stability score comparison. The figure shows adjusted stability scores for feature selection methods CatBoost with SHAP values (GBDT-SHAP), LASSO logistic regression and five different XGBoost (XGB) built-in feature importance calculation methods (weight, gain, cover, total gain, and total cover).

**References**

1 Cristianini, N. & Shawe-Taylor, J. *An introduction to support vector machines and other kernel-based learning methods*. (Cambridge university press, 2000).

2 Fix, E. & Hodges, J. L. Discriminatory analysis. Nonparametric discrimination: Consistency properties. *International Statistical Review/Revue Internationale de Statistique* **57**, 238-247 (1989).

3 McCulloch, W. S. & Pitts, W. A logical calculus of the ideas immanent in nervous activity. *The bulletin of mathematical biophysics* **5**, 115-133 (1943).

4 Rosenblatt, F. The perceptron: a probabilistic model for information storage and organization in the brain. *Psychological review* **65**, 386 (1958).

5 Kim, S. Y. *et al.* A deep learning model for real-time mortality prediction in critically ill children. *Critical Care* **23**, 279 (2019).

6 Meyer, A. *et al.* Machine learning for real-time prediction of complications in critical care: a retrospective study. *The Lancet Respiratory Medicine* **6**, 905-914 (2018).

7 Mohamadlou, H. *et al.* Multicenter validation of a machine-learning algorithm for 48-h all-cause mortality prediction. *Health Informatics Journal*, 1460458219894494 (2019).

8 Weng, S. F., Vaz, L., Qureshi, N. & Kai, J. Prediction of premature all-cause mortality: A prospective general population cohort study comparing machine-learning and standard epidemiological approaches. *PLOS One* **14**, e0214365 (2019).

9 Olson, R. S., Cava, W., Mustahsan, Z., Varik, A. & Moore, J. H. Data-driven advice for applying machine learning to bioinformatics problems. *Pac Symp Biocomput* **23**, 192-203 (2018).

10 Prokhorenkova, L., Gusev, G., Vorobev, A., Dorogush, A. V. & Gulin, A. CatBoost: unbiased boosting with categorical features. *Adv Neur In* **31** (2018).

11 Dorogush, A. V., Ershov, V. & Gulin, A. CatBoost: gradient boosting with categorical features support. *arXiv preprint arXiv:1810.11363* (2018).

12 Bentéjac, C., Csörgő, A. & Martínez-Muñoz, G. A comparative analysis of gradient boosting algorithms. *Artificial Intelligence Review*, 1-31 (2020).

13 Millard, L. A., Davies, N. M., Gaunt, T. R., Davey Smith, G. & Tilling, K. Software Application Profile: PHESANT: a tool for performing automated phenome scans in UK Biobank. *International Journal of Epidemiology* (2017).

14 Shapley, L. S. A value for n-person games. *Contributions to the Theory of Games* **2**, 307-317 (1953).

15 Lundberg, S. M. & Lee, S.-I. A unified approach to interpreting model predictions. *Advances in Neural Information Processing Systems*, 4765-4774 (2017).

16 Lundberg, S. M. & Lee, S.-I. Consistent feature attribution for tree ensembles. *arXiv preprint arXiv:1706.06060* (2017).

17 Tibshirani, R. Regression shrinkage and selection via the lasso. *Journal of the Royal Statistical Society: Series B (Methodological)* **58**, 267-288 (1996).

18 Pedregosa, F. *et al.* Scikit-learn: Machine learning in Python. *the Journal of machine Learning research* **12**, 2825-2830 (2011).

19 Kalousis, A., Prados, J. & Hilario, M. Stability of feature selection algorithms: a study on high-dimensional spaces. *Knowledge and information systems* **12**, 95-116 (2007).

20 Kuncheva, L. I. A stability index for feature selection. *Artificial intelligence and applications*, 421-427 (2007).
